# Biological Evaluation of Arylsemicarbazone Derivatives as Potential Anticancer Agents

**DOI:** 10.3390/ph12040169

**Published:** 2019-11-17

**Authors:** Anne Cecília Nascimento da Cruz, Dalci José Brondani, Temístocles I´talo de Santana, Lucas Oliveira da Silva, Elizabeth Fernanda da Oliveira Borba, Antônio Rodolfo de Faria, Julianna Ferreira Cavalcanti de Albuquerque, Sylvie Piessard, Rafael Matos Ximenes, Blandine Baratte, Stéphane Bach, Sandrine Ruchaud, Francisco Jaime Bezerra Mendonça Junior, Marc-Antoine Bazin, Marcelo Montenegro Rabello, Marcelo Zaldini Hernandes, Pascal Marchand, Teresinha Gonçalves da Silva

**Affiliations:** 1 Departamento de Antibióticos, Centro de Biociências, Universidade Federal de Pernambuco, Recife, PE 50740-520, Brazil; annececilia2006@hotmail.com (A.C.N.d.C.); temistoclesitalo@gmail.com (T.I.d.S.); elizabethfernanda_7@hotmail.com (E.F.d.O.B.); julianna@ufpe.br (J.F.C.d.A.); ximenesrm@gmail.com (R.M.X.); 2 Departamento de Ciências Farmacêuticas, Centro de Ciências da Saúde, Universidade Federal de Pernambuco, Recife, PE 50740-520, Brazil; brondani.dj@gmail.com (D.J.B.); luc.osilva@gmail.com (L.O.d.S.); rodolfo.ufpe@gmail.com (A.R.d.F.); montenegro.rabello@gmail.com (M.M.R.); zaldini@gmail.com (M.Z.H.); 3 Université de Nantes, Cibles et Médicaments des Infections et du Cancer, IICiMed, EA 1155, F-44000 Nantes, France; sylvie.piessard@univ-nantes.fr (S.P.); marc-antoine.bazin@univ-nantes.fr (M.-A.B.); 4 Sorbonne Université, CNRS, USR3151, « Protein phosphorylation and human diseases » Unit, Station Biologique, F-29688 Roscoff, France; baratte@sb-roscoff.fr (B.B.); bach@sb-roscoff.fr (S.B.); sandrine.ruchaud@sb-roscoff.fr (S.R.); 5 Sorbonne Université, CNRS, FR2424, Kinase Inhibitor Specialized Screening Facility - KISSf, Station Biologique, F-29688 Roscoff, France; 6 Laboratory of Synthesis and Drug Delivery, Department of Biological Sciences, State University of Paraiba, João Pessoa, PB 58071-160, Brazil; franciscojbmendonca@yahoo.com.br

**Keywords:** semicarbazones, cytotoxicity, anticancer activity, cell cycle, apoptosis, kinase inhibition

## Abstract

Fourteen arylsemicarbazone derivatives were synthesized and evaluated in order to find agents with potential anticancer activity. Cytotoxic screening was performed against K562, HL-60, MOLT-4, HEp-2, NCI-H292, HT-29 and MCF-7 tumor cell lines. Compounds **3c** and **4a** were active against the tested cancer cell lines, being more cytotoxic for the HL-60 cell line with IC_50_ values of 13.08 μM and 11.38 μM, respectively. Regarding the protein kinase inhibition assay, **3c** inhibited seven different kinases and **4a** strongly inhibited the CK1δ/ε kinase. The studied kinases are involved in several cellular functions such as proliferation, migration, cell death and cell cycle progression. Additional analysis by flow cytometry revealed that **3c** and **4a** caused depolarization of the mitochondrial membrane, suggesting apoptosis mediated by the intrinsic pathway. Compound **3c** induced arrest in G1 phase of the cell cycle on HL-60 cells, and in the annexin V assay approximately 50% of cells were in apoptosis at the highest concentration tested (26 μM). Compound **4a** inhibited cell cycle by accumulation of abnormal postmitotic cells at G1 phase and induced DNA fragmentation at the highest concentration (22 μM).

## 1. Introduction

Cancer is a group of potentially fatal diseases characterized by uncontrolled growth of abnormal cells due to defects in intracellular signaling, being responsible for enormous health costs around the world [1]. Despite the growing advances of medical science in recent years, cancer remains the leading cause of morbidity and mortality across all age groups worldwide. The GLOBOCAN 2018 report indicates that there will be an estimated 18.1 million new cancer cases and 9.6 million cancer deaths in 2018 [2] and according to estimates of the World Health Organization (WHO), 15 million people will die from this disease by 2030 [3].

Several factors contribute to the onset of cancer. These factors may be inherent to the organism, such as age, gender, and genetic inheritance; or external to the organism, such as smoking, inadequate eating habits, sedentary lifestyle, obesity, exposure to mutagens, among others [4].

The main therapeutic approaches used to treat cancer are surgery, radiotherapy and systemic therapy. Such treatments may be used alone or in combination and the form employed depends on the type and location of the tumor, stage of the disease and conditions concerning the patient to be treated [5]. The systemic therapies include hormone therapy [6], targeted therapy [7], immunotherapy and chemotherapy [8]. Chemotherapy is based on the use of cytotoxic agents that disrupt the cell cycle by altering the structure of DNA, inhibiting DNA synthesis, and disturbing microtubules. Cancer cells differ from normal cells because they are self-sufficient in cell division and survival [9]. Thus, chemotherapeutics cause the death of cells by directly interfering with the DNA or key molecules necessary for cell division [8]. However, as this treatment is systemic, its action is not limited to cancer cells and profound side effects occur in the hematopoietic system, in the mucous cells of the gastrointestinal tract, hair follicle and others [10].

Chemotherapy is one of the pillars of cancer treatment, but targeted therapy has been notable to selectively affect cancer cells based on specific molecular characteristics [10]. Antibodies and other molecules capable of altering the action of ligands (growth factors), receptors and intracellular pathways, are used as targeted therapy. This therapy is directed for the purpose of inhibiting dysregulated pathways in order to block multiplication of malignant cells [11].

Protein kinases are important targets in the treatment of cancer because they regulate various signal transduction pathways responsible for critical physiological processes such as cell cycle progression, migration, cell proliferation and differentiation, survival and apoptosis [12]. Deregulation of kinases results in aberrant responses associated with various types of cancer [13]. Therefore, researchers around the world are searching for more potent and selective molecules to specific cellular targets involved in cancer cell signaling.

Semicarbazones represent an important class of chemicals described in the literature with diverse biological properties such as anticonvulsant [14], antimicrobial, antinocipeptive and anti-inflammatory [15], anti-Alzheimer [16], antichagasic [17], antiproliferative, cytotoxic and pro-apoptotic activity [18], among others. The anticancer activity of semicarbazones is also known and some studies describe these compounds as protein kinase inhibitors [19,20].

Semicarbazones are important pharmacophores in the search for new drugs [21]. The possibility of choosing the substituents of the chain, always seeking a better interaction with biological targets, and possible modulation of the lipophilic character of new derivatives, make semicarbazone intermediates important for the design of drugs [22]. In this context, in continuation of the work of our research groups in the development of new antitumor agents [23,24,25,26,27,28,29,30], the present study aimed to synthesize arylsemicarbazone derivatives with the purpose of evaluating their anticancer activity and their inhibition of protein kinases. Although the synthesis of some of these compounds has already been described in the literature [22,31,32,33], the study of anticancer activity is reported for the first time in this work.

Cytotoxic screening was performed against seven cancer cell lines. Among the tested derivatives, seven presented cytotoxicity for at least three cancer cell lines associated with the determination of the corresponding IC_50_ value. The inhibitory activity was also evaluated against a panel of 10 protein kinases. Compounds **3c** and **4a** showed cytotoxicity for all tested cell lines, particularly on HL-60, so both were chosen for further studies of possible mechanisms of action. 

## 2. Results and Discussion

### 2.1. Chemistry

**Scheme 1:**
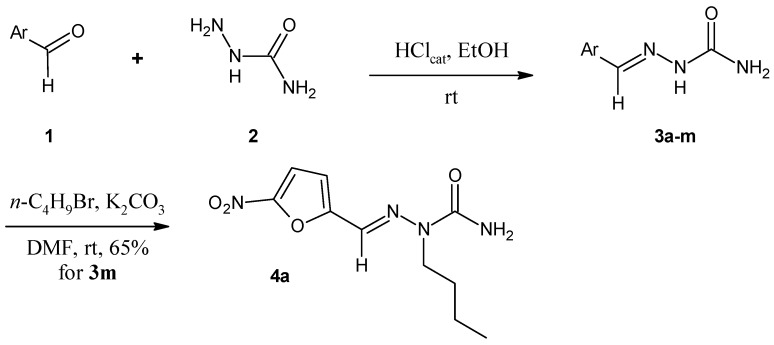
General routes for the synthesis of semicarbazone compounds **3a-m** and **4a**.

The aryl semicarbazone derivatives **3a-3m** were obtained by the classical condensation reaction between suitable aryl and (hetero)aryl aldehydes and semicarbazide in the presence of catalytic hydrochloric acid in ethanol (Scheme 1). Selective one-pot *N*^2^-alkylation of 5-nitro-2-furfuraldehyde semicarbazone **3m** was carried out by nucleophilic substitution on butyl bromide using basic conditions (K_2_CO_3_) in DMF as previously described in the literature by Brondani et al. [22].

The introduction of such a butyl chain would allow us to check the influence of such a lipophilic appendage on biological activities examined within the framework of the study. Indeed, the other analogues of the series remained unsubstituted at the level of this nitrogen.

### 2.2. Cytotoxicity Assays

The cytotoxicity assessment was performed against three human cancer cell lines in suspension (K562, HL-60 and MOLT-4) and four adhered (HEp-2, NCI-H292, HT-29 and MCF-7). Initially, all compounds **3a-3m** and **4a**) were tested in a single concentration of 25 μg/mL. Compounds that exhibited a percentage of inhibition greater than 75% in at least three cell lines were considered active. Among the 14 evaluated derivatives, seven (**3c-3h**, **3m** and **4a**) met this criterion and had their IC_50_ values determined after 72 h of incubation. As shown in Table 1, the derivatives showed different cytotoxicity values.

Only compounds **3c** (2-hydroxynaphthyl) and **4a** (*N*^2^-butyl-5-nitro-2-furfuraldehyde semicarbazone) showed antiproliferative activity against all the tested cell lines, whereas **3a-b**, bearing activating groups (electron-donating groups) and heterocyclic derivatives **3i-l** were inactive. Compound **3m** presented high toxicity on MCF-7 (IC_50_ = 8.56 ± 0.01) and low cytotoxic activity on HL-60, with IC_50_ =24.33 ± 0.16. In addition, cytotoxic activity against some cancer cell lines was observed for halogenated and alkylated compounds **3d-h**.

Compound **3l**, which bears a simple furan, was inactive, while its nitro derivative showed some activity. One possible mechanism of the cytotoxic action of **3m** could be the bioreduction of the nitro group to highly reactive radical intermediates and reactive oxygen species (ROS) generation. This is a mechanism already well described in the anticancer activity of other nitro compounds [34]. However, more specific experiments should be conducted to prove this hypothesis. Interestingly, the counterpart **4a** remained active after the alkylation of **3m** (Table 1).

We next decided to determine the lipophilicity of each tested compound. Indeed, lipophilicity is one of the fundamental factors for the biological activity of drugs because it is directly linked to the degree of permeability of the cell membranes and how much the molecule will be available to interact with the intended biological target(s) [35]. Moreover, solubility was calculated to verify whether compounds were sufficiently water soluble to allow a relevant interpretation for cytotoxicity assays. ClogD_7.4_ and solubility values are shown in Table 2. 

Overall, the predicted solubility obtained for the synthesized compounds were moderate for **3c**, **3f**, **3h** and **4a** (S values between 0.01 and 0.06 mg/mL) and high for all other molecules (S values higher than 0.06 mg/mL). 

Analyzing the aromatic core of the arylsemicarbazones, it was observed that ClogD_7.4_ values span values ranging from −0.07 to 2.87. Notably lipophilicity for compounds **3c-h** and **4a**, higher than 1.5 (i.e. 1.54–2.87), could be considered as a suitable parameter for the active compounds to display enhanced pharmacokinetic properties. 

After discussing the cellular activity against cancer cell lines and the importance of some physicochemical parameters, the data allowed us to identify compounds **3c** and **4a** as the most promising derivatives. These derivatives displayed cytotoxic activity against all the tested cell lines and the best results were against the HL-60 cell line. Compound **3c** showed IC_50_ values ranging from 13.08 to 56.27 μM, while **4a** had IC_50s_ between 11.38 to 37.89 μM, being more cytotoxic for all the panel.

In order to evaluate the cytotoxic potential in normal cells, all the derivatives were tested against Peripheral Blood Mononuclear Cells (PBMCs) and the most promising compounds were again compounds **3c** and **4a,** which offered more selectivity against the cancer cells. The selectivity indexes (SI = IC_50_ PBMC/ IC_50_ cancer cells) of derivatives **3c** and **4a** for the cell line with the lowest IC_50_ (HL-60) were 6.2 and 4.5, respectively. 

### 2.3. Inhibition of Protein Kinases

The eight most cytotoxic compounds (**3c-3h**, **3m** and **4a**) were tested in vitro against a panel of ten different protein kinases known to be involved in the process of initiation and progression of cancer. The protein kinases studied were: CDK2/CyclinA, CDK5/p25, CDK9/CyclinT (cyclin-dependent kinases), HASPIN (haploid germ cell-specific nuclear protein kinase), PIM1 (PIM family kinase 1), CLK1 (cdc2-like kinase 1), DYRK1A (dual-specificity tyrosine phosphorylation regulated kinase), CK1δ/ε (casein kinase 1 isoforms), GSK3α/β (glycogen synthase kinase 3) and AURKB (aurora kinase B). Initially, all compounds were first tested at 10 μM. Compounds displaying less than 50% inhibition were considered as inactive (IC_50_ > 10 μM). Compounds displaying more than 50% inhibition at 10 μM were next tested over a wide range of concentrations (usually 0.01–10 μM), and IC_50_ values were determined from the dose-response curves (Sigma-Plot). The results are shown in Table 3.

Compound **3c** was the most active kinase inhibitor, with IC_50_ values ranging from 0.073–5.1 μM, except towards HASPIN, CK1 and GSK3 kinases. Compound **3c** showed inhibitory activity against seven protein kinases and the highest activity was against DYRK1A. The other counterparts remained inactive in this assay. Compound **4a** exhibited a selective submicromolar IC_50_ of 0.76 μM against the CK1δ/ε isoforms. More specifically, in comparison with other compounds, the presence of a H-donor group in position 2 of the naphthalene ring (compound **3c**) could display efficient interactions with the tested kinases.

### 2.4. Molecular Modeling

For the docking study, three kinases were chosen according to the results of the in vitro assays: tyrosine phosphorylation regulated kinase 1A (DYRK1A), in which compound **3c** was active in the nanomolar range, casein kinase 1 (CK1δ/ε), agianst which compound **4a** was active, and CDK2 for its importance in the cell cycle. Several CDK2 inhibitors have been developed and some of them (including roscovitine, CYC065, dinaciclib, AT7519, milciclib) have undergone clinical evaluation [36]. On the other hand, with dual specificity, DYRK1A plays a key role in Down syndrome and Alzheimer’s disease. In addition, DYRK1A has been shown to be expressed ubiquitously, and its functional role in cancer is still largely obscure, however several compounds caused inhibition of this kinase in HL-60 cells, decreasing its proliferation in vitro [37].

**Figure 1:**
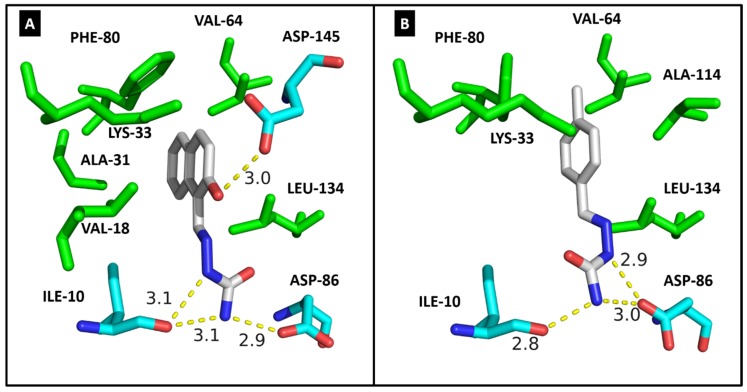
Detailed view of the docking pose for (**A**) compound **3c** and (**B**) compound **3g**, regarding the target CDK2. Residues involved in hydrophobic interactions (green), hydrogen bonds (colored per element with carbon atoms in cyan) are highlighted.

In order to elucidate how the **3c** molecule interacts with CDK2, a detailed docking study was conducted, also including compound **3g** for comparison reasons. Figure 1A illustrates the docking pose for **3c** and Figure 1B illustrates the docking pose for **3g**. As can be seen in Figure 1, the molecular reasons for the greater stability (greater docking score) or affinity of **3c** ligand in the active site of CDK2 can be explained mainly by the presence of important intermolecular interactions, in particular one hydrogen bond that the hydroxyl group in position 2 of the naphthalene ring of compound **3c** additionally establishes with the residue ASP-145 (3.0 Å), when compared with molecule **3g** (Figure 1B). A detailed comparison between the docking results obtained for these two ligands can be found in Table 4. The fact that the molecule **3c** has a greater docking score in comparison with the molecule **3g**, i.e., a greater in silico affinity for the target, correlates with the higher in vitro activity of the molecule **3c** (see Table 3), or the higher inhibition capacity of the molecule **3c** on this target (CDK2).

**Figure 2:**
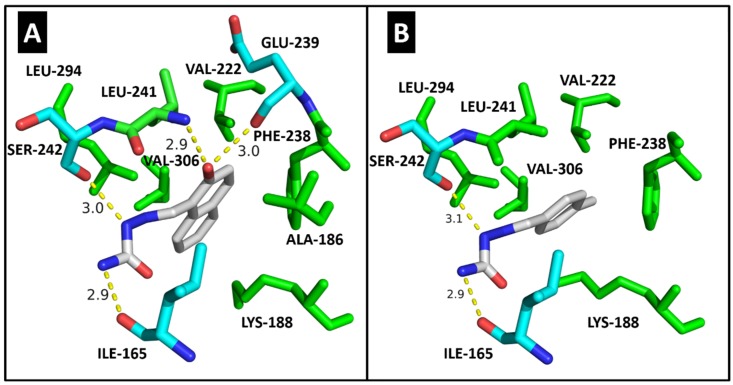
Detailed view of the docking pose for (**A**) compound **3c** and (**B**) compound **3g**, regarding DYRK1A target. Residues involved in hydrophobic interactions (green), hydrogen bonds (colored per element with carbon atoms in cyan) and the involved in both interaction (colored per element with carbon atoms in green) are highlighted.

Additionally, docking of compound **3c** was made on two of the six kinases, and a comparison with compound **3g** was done. This study supported the fact that **3c** inhibits DYRK1A well and better than **3g** (see Table 3). Figure 2A illustrates the docking pose for **3c** and Figure 2B illustrates the docking pose for **3g**. The molecular reasons for the greater stability of the **3c** ligand in the active site of DYRK1A can be explained mainly by the presence of two additional hydrogen bonds that the hydroxyl group in position 2 of the naphthalene ring of compound **3c** establishes with the residues GLU-239 (3.0 Å) and LEU-241 (2.9 Å), when compared with molecule **3g** (Figure 2B). A detailed comparison between the docking results obtained for these two ligands, regarding the DYRK1A target, can also be found in Table 4.

The fact that DYRK1A has a greater docking score in comparison with CDK2 target, regarding compound **3c**, agrees with the in vitro assay (see Table 3) results and can be explained by the shorter hydrogen bonds and the greater amount of hydrophobic contacts observed in the complex formed by compound **3c** and the DYRK1A enzyme. It is also important to highlight that the hydroxyl group in position 2 of the naphthalene ring of compound **3c** establishes at least one hydrogen bond with the CDK2 and DYRK1A kinases.

**Figure 3:**
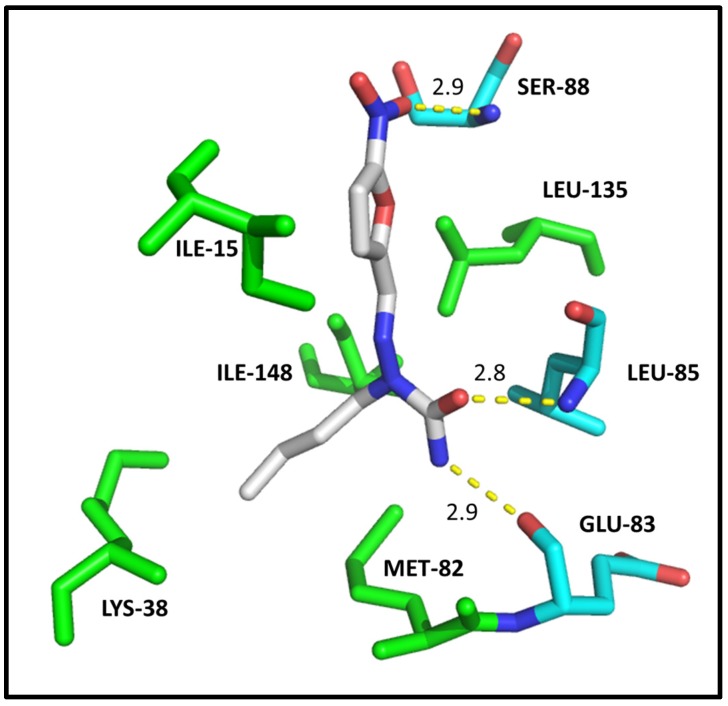
Detailed view of the docking pose for compound **4a** regarding the CK1δ/ε target. Residues involved in hydrophobic interactions (green) and hydrogen bonds (colored per element with carbon atoms in cyan) are highlighted.

According to in vitro assay, compound **4a** is active only at the CK1δ/ε target, therefore docking analysis was performed in order to elucidate the molecular reasons of his potency (0.76 μM). It can be explained mainly by the presence of three hydrogen bonds and by five residues interacting through hydrophobic contacts, as can be seen at Figure 3 and Table 4. 

Protein kinases are involved in several cellular functions, such as gene transcription, cell cycle progression, cytoskeletal rearrangement, proliferation, differentiation, migration, and cell death [38,39]. Deregulation of the signaling pathways of these proteins is associated with several diseases, including cancer [40,41]. Consequently, this class of enzymes represents important therapeutic targets for treatment of diseases [42]. 

In the literature some semicarbazone derivatives are reported as protein kinase inhibitors [19,20]. Thus, this information motivated the accomplishment of the kinase testing that indicated **3c** as a promising compound for further studies to determine the possible mechanism of action. Compound **4a** was also included, because it was active for all the tested cancer cell lines, and presented CK1δ/ε kinase inhibitory activity, of interest because casein kinase 1δ/ε has been identified as a promising therapeutic target for oncology application.

### 2.5. Detection of Cell Viability Using the Guava ViaCount Kit

**Figure 4:**
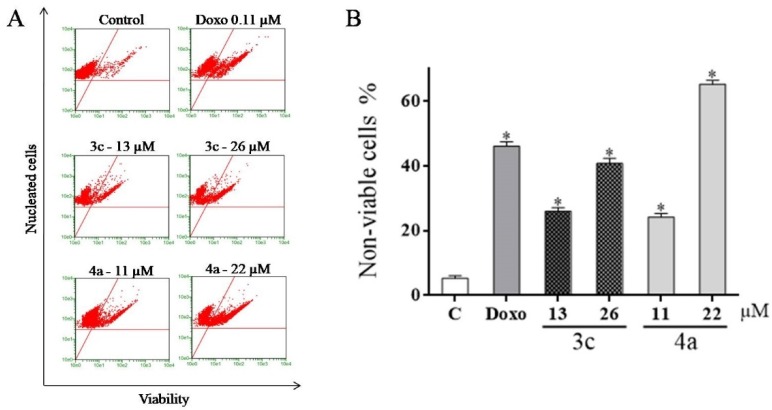
Effect of compounds **3c** and **4a** on cell viability of HL-60 cells using the ViaCount kit after 72 h of incubation. (**A**) Dot plots, where viable cells are located in the upper left quadrant, and non-viable cells in the upper right quadrant; (**B**) The percentage of non-viable cells was shown as the mean ± SD from three independent experiments performed in triplicate. * *p* < 0.05 compared with the negative control by ANOVA followed by Student Newman-Keuls test. C = control; Doxorubicin (Doxo, 0.11 μM) was used as a standard drug. The negative control was treated with the vehicle (DMSO; 0.1%).

Initially, the percentage of viable cells in apoptosis or necrosis was determined by the ViaCount assay at the Guava EasyCyte HT flow cytometer (Merck-Millipore, Hayward, CA, USA). The ViaCount kit (Merck-Millipore, Hayward, CA, USA) determines cell viability based on membrane permeability. The concentrations used in the cytometric tests were values corresponding to the IC_50_ and 2x IC_50_ of 72 h and the chosen cell line was HL-60 (promyelocytic leukemia). The results showed that the two compounds significantly decreased cell viability (Figure 4).

At the two concentrations tested, **3c** increased the number of cells in apoptosis. Compound **4a** increased the number of cells in early apoptosis, mainly at the concentration of 22 μM, where 65.3% of the cells were in early apoptosis. Doxorubicin increased the number of cells in apoptosis and that died.

### 2.6. Evaluation of Mitochondrial Transmembrane Potential (ΔΨm)

Mitochondria play an essential role in the life and death of cells, as they are responsible for the energy production necessary for cell survival and also regulate apoptosis. The good performance in energy production and the integrity of the mitochondria are guaranteed by the maintenance of mitochondrial electrical potential [43]. Some drugs act by inducing the loss of mitochondrial transmembrane potential leading to a process called mitochondrial depolarization, which is one of the early events in the process of cell death by apoptosis triggered by the intrinsic (mitochondrial) pathway [44].

**Figure 5:**
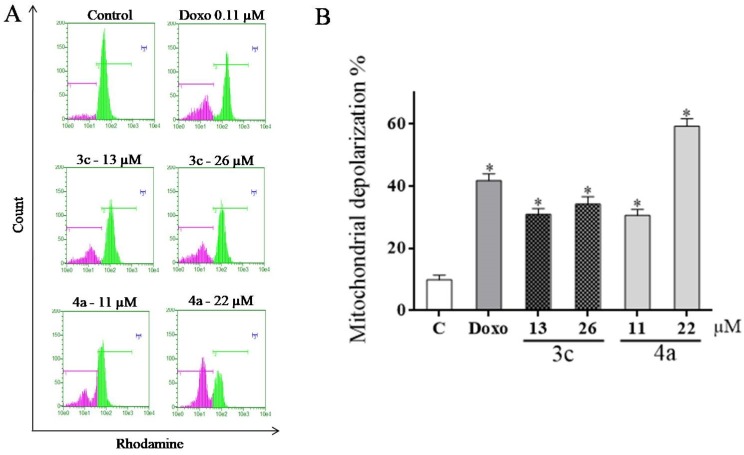
Effect of compounds 3c and 4a on the mitochondrial transmembrane potential (ΔΨm) of HL-60 cells after 72 h of incubation. (**A**) Depolarized cells (apoptotic) are stained in purple, while non-depolarized cells (non-apoptotic) are stained in green. (**B**) The percentage of cells with depolarized mitochondrial membrane (apoptotic cells). C = Control, cells were treated with the vehicle (DMSO; 0.1%); Doxorubicin (Doxo, 0.11 μM) was used as a standard drug. Results are expressed as mean ± SD of at least three different experiments performed in triplicate. * *p* < 0.05 compared with the negative control by ANOVA followed by Student Newman-Keuls test.

In this sense, in order to evaluate if compounds **3c** and **4a** induce apoptosis by alterating the mitochondria transmembrane potential, this assay was performed by flow cytometry using the fluorochrome rhodamine 123, because this is able to accumulate in cells with unchanged mitochondrial transmembrane potential. After 72 h of incubation, **3c** and **4a** were found to be able of inducing mitochondrial depolarization in HL-60 cells (Figure 5).

Cells treated with **3c** at concentration values of 13 and 26 μM produced 30.9% and 34.2% of depolarized cells, respectively. These data suggest the cell death caused by **3c** involves other death pathways beyond the intrinsic mitochondrial pathway of apoptosis. On the other hand, compound **4a** was more active and induced depolarization in 59.3% of the cells at a concentration of 22 μM. The positive control, doxorubicin, led to 41.8% of depolarized cells. 

Apoptosis is a key programmed cell-death pathway involved in numerous processes. One of them is the balance between cell proliferation and death, essential for the maintenance of tissue homeostasis. In general, two major signaling pathways control apoptosis: (i) mitochondria-mediated or intrinsic pathway and (ii) death receptor-mediated or extrinsic pathway [45]. When the cell undergoes pro-apoptotic stimuli, such as deprivation of growth factors, DNA damage, hypoxia, activation of oncogenes, among others, the signals that are translated converge mainly to mitochondria causing the collapse of the potential of the internal mitochondrial membrane (Δψm) that trigger death by apoptosis [46]. The results obtained in this test demonstrated that the compounds elicited pro-apoptotic effects that induced mitochondrial depolarization in HL-60 cells.

### 2.7. Cell Cycle Assay

**Figure 6:**
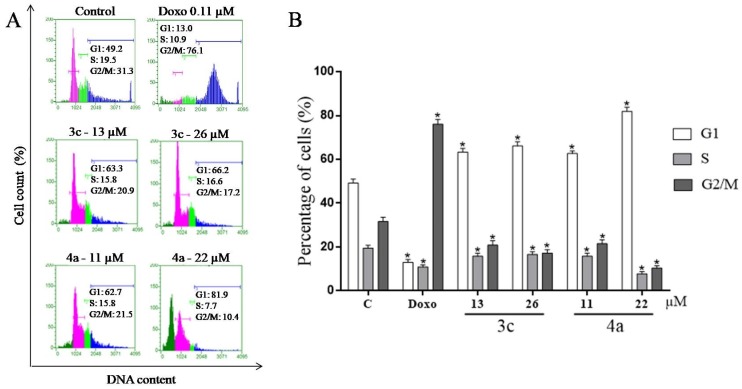
Effect of compounds **3c** and **4a** on the cell cycle of HL-60 cells after 72 h of incubation. A) (**A**) Cell cycle analysis was performed using flow cytometry and representative histograms on different colors representing show the distribution of cells in the G0/G1, S and G2/M phase. (**B**) Summary histograms indicating the percentage of cells in each phase are presented. Doxorubicin (Doxo, 0.11 μM) was used as a standard drug. C = control, the cells were treated with the vehicle (DMSO; 0.1%). Results are expressed as mean ± SD of at least three different experiments performed in triplicate. * *p* < 0.05 compared with the negative control by ANOVA followed by Student Newman-Keuls test.

In order to improve the study of the mechanism of death induction by compounds **3c** and **4a** in HL-60 cells, a test was performed on the flow cytometer after staining with propidium iodide to evaluate the effect of the compounds on cell cycle progression (Figure 6).

After the treatment period with **3c** (13 μM), 63.3% of the cells were in the G1 phase. At the concentration of 26 μM, the population in the G1 phase increased to 66.2%. These results indicate that **3c** causes cell cycle arrest in the G1 phase. 

Treatment with **4a** increased the cell population in the G1 phase by 62.7% (11 μM) and 81.9% (22 μM), respectively, indicating cell cycle arrest in the G1 phase. In the histogram (**4a**; 22 μM) it is possible to visualize large amounts of cell debris. The chemotherapeutic doxorubicin caused arrest at G2/M. The cell cycle is the process by which cells multiply. This is divided into four phases: G1, S, G2 and M. To ensure correct progression through the cell cycle, cells have developed checkpoints that are mechanisms that evaluate cell conditions before beginning the next phase, thereby ensuring cell division fidelity [47]. At these checkpoints, if necessary, cell cycle arrest occurs and DNA repairs are performed, however, depending on the efficacy of the process, the cycle is not completed and the cell is referred for apoptosis. The regulation of the cell cycle is a balance between the positive regulators which induce cell replication and negative regulators that prevent replication [48].

Positive regulators are formed by protein kinases that act together stimulating the continuity of the cycle [49]. Previously, CDK5 was thought to function in a cell cycle independent manner, however, recently the retinoblastoma protein (Rb) was discovered as a downstream target of CDK5. Expression of CDK5 leads to the phosphorylation of Rb, ultimately leading to the expression of cyclins and others cdks [50]. In the protein kinase inhibition test, it was found that **3c** inhibited the CDK5/p25 complex, probably preventing the phosphorylation of pRb protein, preventing the cell leaving the G1 stage and entering the S phase. The stop of the cell cycle in G1 caused by **3c** corroborates with this hypothesis.

According to Huart et al. [51], more than four hundred proteins interact with p53 and many of them act as inhibitors. Studies have shown that the casein kinase 1 (CK1) family phosphorylates the MDM2 protein and modulates the interaction with p53 under different conditions. Inuzuka et al. [52] observed that under normal conditions, the CK1δ/ɛ isoforms are able to phosphorylate several serine residues in the acid domain of MDM2 that are involved in the degradation of p53, acting as a negative regulator of this protein.

Studies suggest that dysregulation of these cellular processes contributes to oncogenesis, and that pharmacological inhibition of CK1 can increase p53 protein levels in cells and induce cell death. Thus, the inhibition of these kinases represents an important approach in anticancer therapy [53,54].

Compound **4a** inhibited CK1δ/ε isoform activity. That result may be associated with cell cycle arrest at G1 checkpoint caused by **4a** in HL-60 cells, since CK1δ/ε inhibition prevents the transition of abnormal postmitotic cells from G1 to S phase [55].

### 2.8. DNA Fragmentation Assay

Cell death by apoptosis is characterized by marked morphological changes such as chromatin condensation, DNA fragmentation, cell membrane blebbing and formation of apoptotic bodies [56].

**Figure 7:**
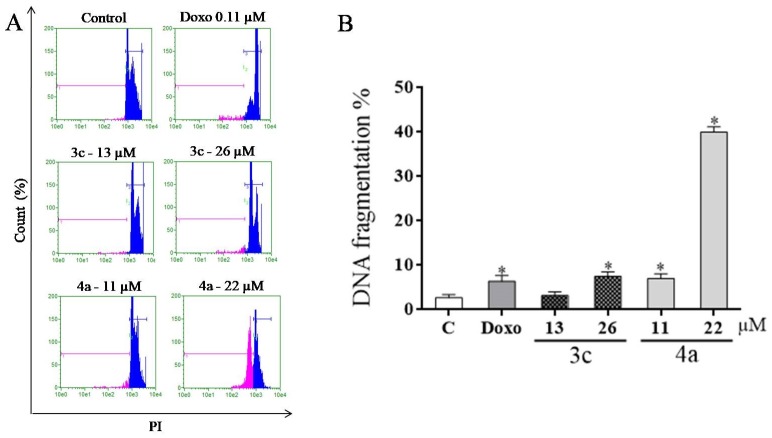
Effect of compounds **3c** and **4a** on DNA fragmentation of HL-60 cells after 72 h of incubation. (**A**) Apoptotic cells with fragmented DNA (pink color) are identified as the sub-G1 population, seen to the left of the G1 peak (blue color). (**B**) Histograms indicating the percentage of cells with DNA fragmentation. Doxorubicin (Doxo, 0.11 μM) was used as standard. C = negative control treated with the vehicle (DMSO; 0.1%). Results are expressed as mean ± SD of at least three different experiments performed in triplicate. * *p* < 0.05 compared with the negative control by ANOVA followed by Student Newman-Keuls test.

DNA fragmentation is initiated after chromatin condensation and depends on several variables, such as the time required for the depolarization of the mitochondria and activation of the caspase cascade. In response to DNA damage the p53 protein is activated causing cell cycle arrest in G1 [57]. At flow cytometry the apoptotic cells with fragmented DNA can be identified as the sub-G1 population, seen to the left of the G1 peak [58]. Thus, using the fluorochrome propidium iodide and the same principle of the cell cycle test, an assay was performed to identify HL-60 cells with fragmented DNA (Figure 7).

Compound **3c** induced fragmentation in 3.2% and 7.5% of cells at concentrations of 13 μM and 26 μM, respectively. Compound **4a** induced fragmentation in 7.0% and 40.3% of the cells at concentrations of 11 and 22 μM, respectively. This result corroborates the inhibition of CK1δ/ε and the accumulation of abnormal postmitotic cells at G1 phase [55].

### 2.9. Study of the Induction of Cell Death by the Annexin V Test

**Figure 8:**
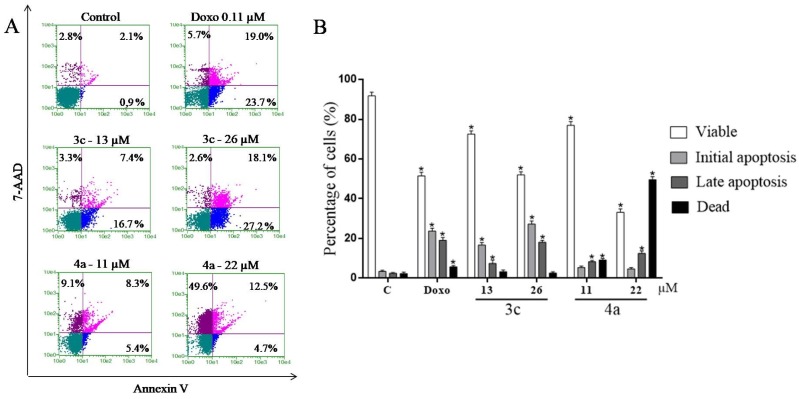
Effect of compounds **3c** and **4a** on the induction of death of HL-60 cells after 72 h of incubation. (**A**) Distribution of cells positive for Annexin V and/or 7-AAD is reported inside the quadrants. (**B**) The percentage of viable, apoptotic and dead cells. Doxorubicin (Doxo, 0.11 μM) was used as standard. C = control, was treated with the vehicle (DMSO; 0.1%). Results are expressed as mean ± SD of at least three different experiments performed in triplicate. * *p* < 0.05 compared with the negative control by ANOVA followed by Student Newman-Keuls test.

Aiming to better study the mechanism of induction of death involved in HL-60 cells after treatment with compounds **3c** and **4a**, a flow cytometric test was performed after double staining with annexin V-FITC/7-AAD. This assay is based on the ability of annexin V to bind with high affinity to phosphatidylserine which is translocated to the outer membrane of apoptotic cells and the 7-AAD dye binds to the DNA of cells with loss of membrane integrity. Thus, in this test it is possible to identify four distinct populations: viable cells [Annexin V(−) and 7-AAD(−)], initial apoptotic cells [Annexin V(+) and 7-AAD(−)], late apoptotic cells [Annexin V(+) and 7-AAD(+)] and dead cells [Annexin V(−) and 7-AAD (+)] [59]. The results are shown in Figure 8.

After treatment with **3c** (13 and 26 μM), it was observed that 16.7 and 27.2% of the cells were in initial apoptosis and 7.4 and 18.1% in late apoptosis phase, respectively. In the treatment with **4a** (11 μM) 5.4% of the cells were in initial apoptosis, 8.3% in late apoptosis and 9.1% were dead. Cells treated with **4a** (22 μM) showed 4.7% of the cells in initial apoptosis, 12.5% in late apoptosis and almost 50% of the cells were dead. Regarding the treatment with doxorubicin, 23.7% of the cells were in initial apoptosis and 19% in late apoptosis.

In cell death by apoptosis, the cell undergoes morphological changes striking and coordinated. Initially, the cellular plasma membrane remains intact but undergoes structural changes, such as the distribution of phosphatidylserine in the outer layer of the membrane, which is a signal for recognition by phagocytes. Thus, the dead cell is rapidly phagocytosed by macrophages and removed without extravasation of the cytoplasmic content [60].

The main death mode caused by **3c** was apoptosis, confirmed in all the tests. The action of **3c** on cell death by apoptosis is probably related to strong inhibition of protein kinases, which causes cell cycle arrest and triggers pro-apoptotic signals that lead to the collapse of mitochondrial membrane potential, culminating in apoptosis.

Derivative **4a**, at the highest concentration (22 μM), showed strong cytotoxicity and caused DNA fragmentation in HL-60 cells. The compound **4a** is a nitro derivative and the effect can be associated with the mechanism of action of this class of substances that is related to the reduction of the nitro group, resulting in reactive species that interact with the plasma membrane of the cells and with essential biomolecules, causing the cytotoxic effect. However, further studies need to be done to elucidate the mechanism of action of this compound. 

## 3. Materials and Methods 

### 3.1. Chemistry 

#### 3.1.1. General Experimental Procedures

All melting points were determined using a Fisatom 430D apparatus (Fisatom, São Paulo, Brazil) and are uncorrected. FTIR spectra were obtained on a model IFS66 spectrophotometer (Bruker, Billerica, MA, USA) using KBr pellets. NMR spectra were recorded on Bruker Avance 400 spectrometer (^1^H-NMR at 400 MHz and ^13^C-NMR at 100 MHz), the residual signal of the solvent was used as internal reference. Chemical shift (δ) values are expressed in parts per million (ppm) and coupling constants (*J*) values are given in Hz. Abbreviations: s = singlet; d = doublet; t = triplet; q = quadruplet; m = multiplet. Elemental analyses were performed on a PE-2400 instrument (PerkinElmer Inc., Waltham, MA, USA) and the results were within acceptable range. Thin layer chromatography (TLC) was carried out on silica gel plates with a fluorescence indicator of F254 (0.2 mm, E. Merck, Darmstadt, Germany). All reagents used in the present study were of analytical grade and were purchased from Aldrich or Fluka (both São Paulo, Brazil) and were used without additional purification.

#### 3.1.2. General Procedure for the Preparation of Arylsemicarbazones **3a**–**3m**

Compounds **3a-3i** and **3k-3m** were obtained by reaction of 2.5 mmol of semicarbazide hydrochloride and an equimolar amount of the appropriate aryl aldehyde in 15 mL of absolute ethanol as solvent. Three drops of hydrochloric acid were added to the medium as catalyst. The reaction was kept at room temperature and under constant magnetic stirring for a period of 3–5 h. To obtain derivative **3j**, 5 mmol of semicarbazide hydrochloride (two equivalents) were added to 2.5 mmol of the respective aldehyde. The reactions were monitored by means of thin layer chromatography and, upon completion, the arylsemicarbazone precipitated in the reaction medium was filtered in sintered funnel and washed with water, followed by washing in hexane. Purification of the synthesized compounds was performed by recrystallization from distilled water. 

##### 4-Methoxybenzaldehyde Semicarbazone (**3a**)

M.F.: C_9_H_11_N_3_O_2_, M.W.: 193.20, White solid; yield: 82%; m.p.: 210–212 °C [31]; Rf: 0.53 (ethyl acetate). ^1^H-NMR: DMSO-d_6_ (δ ppm): 3.78 (s, 3H, OCH_3_); 6.42 (s, 2H, NH_2_); 6.93 (d, 2H, *J* = 8.80 Hz, Ar-H3,5); 7.64 (d, 2H, *J* = 8.80 Hz, Ar-H2,6); 7.78 (s, 1H, CH=N); 10.10 (s, 1H, NH). ^13^C-NMR, DMSO-d_6_ (δ ppm): 159.98, 156.78, 139.13, 127.97 (2C), 127.40, 114.01 (2C), 55.16. Anal. Calcd.: C, 55.95; H, 5.75. Found: C, 56.12; H, 5.89.

##### 4-Hydroxy-3-methoxybenzaldehyde Semicarbazone (**3b**)

M.F.: C_9_H_11_N_3_O_3_ M.W.: 209.20, yellow solid; yield: 81%; m.p.: 237–239 °C [32]; Rf: 0.43 (ethyl acetate). ^1^H-NMR, DMSO-d_6_ (δ ppm): 3.81 (s, 3H, CH_3_); 6.45 (s, 2H, NH_2_); 6.75 (d, 1H, *J* = 7.79 Hz, Ar-H5); 6.96 (d, 1H, *J* = 7.79 Hz, *J* = 1.20 Hz, Ar-H6); 7.37 (d, 1H, *J* = 1.20 Hz, Ar-H2); 7.71 (s, 1H, CH=N); 9.29 (s, 1H, OH); 10.03 (s, 1H, NH). ^13^C-NMR, DMSO-d_6_ (δ ppm): 157.02, 148.14, 148.11, 139.92, 126.49, 121.16, 115.34, 109.15, 55.84. Anal. Calcd.: C, 51.67; H, 5.29. Found: C, 51.77; H, 5.36.

##### 2-Hydroxynaphthaldehyde Semicarbazone (**3c**)

F.M.: C_12_H_11_N_3_O_2_ M.W.: 229.23 yellow solid; yield: 82%; m.p.: 220–223 °C; Rf: 0.59 (ethyl acetate). RMN ^1^H NMR: DMSO-d_6_ (δ ppm): 6.40 (s, 2H, NH_2_); 7.19 (d, 1H, J = 8.99, Ar-H); 7.37 (t, 1H, *J* = 7.49, Ar-H); 7.55 (t, 1H, *J* = 7.49, Ar-H); 7.83 (d, 2H, *J* = 7.49, Ar-H); 8.37 (d, 1H, *J* = 8.99, Ar-H); 8.87 (s, 1H, CH=N); 10.25 (s, 1H, NH); 11.20 (bs, 1H, OH). ^13^C NMR, DMSO-d_6_ (δ ppm): 156.26, 156.00, 140.05, 131.57, 131.51, 128.84, 128.13, 127.64, 123.43, 122.15, 118.63, 109.98. Anal. Calcd.: C, 62.87; H, 4.83. Found: C, 62.79; H, 4.96.

##### 4-Bromobenzaldehyde Semicarbazone (**3d**)

F.M.: C_8_H_8_BrN_3_O M.W.: 242.07 white solid; yield: 83%; m.p.: 237–239 °C [31]; Rf: 0.64 (ethyl acetate). ^1^H-NMR: DMSO-d_6_ (δ ppm): 6.54 (s, 2H, NH_2_); 7.56 (d, 2H, *J* = 6.59 Hz, Ar-H); 7.69 (d, 2H, *J* = 6.59 Hz, Ar-H); 7.79 (s, 1H, CH=N), 10.33 (s, 1H, NH). ^13^C-NMR, DMSO-d_6_ (δ ppm): 156.58, 137.87, 134.10, 131.44 (2C), 128.40 (2C), 121.99. Anal. Calcd.: C, 39.69; H, 3.33. Found: C, 39.87; H, 3.29.

##### 4-Chlorobenzaldehyde Semicarbazone (**3e**)

F.M.: C_8_H_8_ClN_3_O M.W.: 197.62 white solid; yield: 81%; m.p.: 235–236 °C [32]; Rf: 0.65 (ethyl acetate). ^1^H-NMR: DMSO-d_6_ (δ ppm): 6.55 (s, 2H, NH_2_); 7.42 (d, 2H, *J* = 8.39 Hz, Ar-H); 7.75 (d, 2H, *J* = 8.39 Hz, Ar-H); 7.81 (s, 1H, CH=N); 10.32 (s, 1H, NH). ^13^C-NMR, DMSO-d_6_ (δ ppm): 156.57, 137.77, 133.76, 133.27, 128.53 (2C), 128.13 (2C). Anal. Calcd.: C, 48.62; H, 4.08. Found: C, 48.71; H, 4.13.

##### 3,4-Dichlorobenzaldehyde Semicarbazone (**3f**)

F.M.: C_8_H_7_Cl_2_N_3_O M.W.: 232.06 white solid; yield: 88%; m.p.: 246–247 °C [31]; Rf: 0.60 (ethyl acetate). ^1^H NMR: DMSO-d_6_ (δ ppm): 6.67 (s, 2H, NH_2_); 7.65 (d, 1H, *J* = 8.00 Hz, Ar-H5); 7.71 (dd, 1H, *J* = 8.00 Hz, *J* = 1.60 Hz, Ar-H6); 7.82 (s, 1H, CH=N); 8.16 (d, 1H, *J* = 1.60 Hz, Ar-H2); 10.45 (s, 1H, NH). ^13^C NMR, DMSO-d_6_ (δ ppm): 156.55, 136.40, 135.70, 131.59, 130.91, 130.60, 127.61, 126.81. Anal. Calcd.: C, 41.40; H, 3.04. Found: C, 41.56; H, 3.12.

##### 4-Methylbenzaldehyde Semicarbazone (**3g**)

F.M.: C_9_H_11_N_3_O M.W.: 177.20 white solid; yield: 82%; m.p.: 215–217 °C [33]; Rf: 0.51 (ethyl acetate). ^1^H-NMR: DMSO-d_6_ (δ ppm): 2.31 (s, 3H, CH_3_); 6.43 (s, 2H, NH_2_); 7.19 (d, 2H, *J* = 8.00 Hz, Ar-H3,5); 7.59 (d, 2H, *J* = 8.00 Hz, Ar-H2,6); 7.80 (s, 1H, CH=N); 10.16 (s, 1H, NH). ^13^C-NMR, DMSO-d_6_ (δ ppm): 156.68, 139.28, 138.54, 132.03, 129.11 (2C), 126.44 (2C), 20.89. Anal. Calcd.: C, 61.00; H, 6.25. Found: C, 61.23; H, 6,28.

##### 4-Tert-Butylbenzaldehyde Semicarbazone (**3h**)

F.M.: C_12_H_17_N_3_O M.W.: 219.28 white solid; yield: 92%; m.p.: 216–218 °C; Rf: 0.57 (ethyl acetate). ^1^H-NMR: DMSO-d_6_ (δ ppm): 1.28 (s, 9H, (CH_3_)_3_); 6.44 (s, 2H, NH_2_); 7.39 (d, 2H, *J* = 8.69 Hz, Ar-H); 7.62 (d, 2H, *J* = 8.69 Hz, Ar-H); 7.81 (s, 1H, CH=N); 10.19 (s, 1H, NH). ^13^C-NMR, DMSO-d_6_ (δ ppm): 156.67, 151.64, 139.18, 132.03, 126.26 (2C), 125.27 (2C), 34.41, 30.95 (3C). Anal. Calcd.: C, 65.72; H, 7.81. Found: C, 65.59; H, 7.98.

##### 4-Pyridinecarboxaldehyde Semicarbazone (**3i**)

F.M.: C_7_H_8_N_4_O M.W.: 164.16 white solid; yield: 66%; m.p.: 225–226 °C; Rf: 0.84 (chloroform/methanol 7:3). ^1^H-NMR: DMSO-d_6_ (δ ppm): 6.67 (s, 2H, NH_2_); 7.69 (d, 2H, *J* = 4.80 Hz, Ar-H3-5); 7.80 (s, 1H, CH=N); 8.55 (d, 2H, *J* = 4.80 Hz, Ar-H2-6); 10.59 (s, 1H, NH). ^13^C-NMR, DMSO-d_6_ (δ ppm): 156.62, 150.09 (2C), 142.16, 136.72, 120.76 (2C). Anal. Calcd.: C, 51.21; H, 4.91. Found: C, 51.32; H, 4.89.

##### 2,6-Pyridinedicarboxaldehyde Semicarbazone (**3j**)

M.F.: C_9_H_11_N_7_O_2_ M.W.: 249.23 yellow solid; yield: 55%; m.p: 270–272 °C [32]; Rf: 0.66 (chloroform/methanol 1:1). ^1^H-NMR: DMSO-d_6_ (δ ppm): 6.63 (s, 4H, NH_2_); 7.79 (t, 1H, *J* = 7.80 Hz, Ar-H4); 7.85 (s, 2H, CH=N); 8.07 (d, 2H, *J* = 8.00 Hz, Ar-H3-5); 10.53 (s, 2H, NH). ^13^C-NMR, DMSO-d_6_ (δ ppm): 156.61 (2C), 153.53 (2C), 139.57 (2C), 136.86, 119.23 (2C). Anal. Calcd.: C, 43.37; H, 4.44. Found: C, 43.23; H, 4.48.

##### 2-Thiophenecarboxaldehyde Semicarbazone (**3k**)

M.F.: C_6_H_7_N_3_OS M.W.: 169.20, clear yellow solid; yield: 84%; m.p.: 227–230 °C; Rf: 0.54 (ethyl acetate). ^1^H-NMR: DMSO-d_6_ (δ ppm): 6.27 (s, 2H, NH_2_); 7.07 (dd, 1H, *J* = 3.59 Hz, *J* = 5.09 Hz, Ar-H4); 7.31 (d, 1H, *J* = 3.59 Hz, Ar-H3); 7.55 (d, 1H, *J* = 5.09 Hz, Ar-H5); 8.03 (s, 1H, CH=N); 10.24 (s, 1H, NH). ^13^C-NMR, DMSO-d_6_ (δ ppm): 156.46, 139.59, 134.96, 128.86, 127.80, 127.53. Anal. Calcd.: C, 42.59; H, 4.16. Found: C, 42.53; H, 4.19.

##### 2-Furfuraldehyde Semicarbazone (**3l**)

M.F.: C_6_H_7_N_3_O_2,_ M.W.: 153.14, off-white solid; yield: 72%; m.p.: 202–204 °C [31]; Rf: 0.49 (ethyl acetate). ^1^H-NMR: DMSO-d_6_ (δ ppm): 6.32 (s, 2H, NH_2_); 6.57 (dd, 1H, *J* = 2.99 Hz, *J* = 3.59 Hz, Ar-H4); 6.81 (d, 1H, *J* = 3.59 Hz, Ar-H3); 7.74 (d, 1H, *J* = 2.99 Hz, Ar-H5); 7.76 (s, 1H, CH=N); 10.23 (s, 1H, NH). ^13^C-NMR, DMSO-d_6_ (δ ppm): 156.58, 150.05, 144.16, 130.05, 112.09, 110.99. Anal. Calcd.: C, 47.05; H, 4.60. Found: C, 47.23; H, 4.62.

##### 5-Nitro-2-furfuraldehyde Semicarbazone (**3m**)

M.F.: C_6_H_6_N_4_O_4,_ M.W.: 198.14, yellow solid; yield: 70%; m.p.: 190–191 °C [22]; Rf: 0.51 (ethyl acetate). ^1^H-NMR: DMSO-d_6_ (δ ppm): 6.61 (s, 2H, NH_2_); 7.23 (d, 1H, *J* = 3.60 Hz, Ar-H3); 7.78 (d, 1H, *J* = 3.60 Hz, Ar-H4); 7.79 (s, 1H, CH=N); 10.79 (s, 1H, NH). ^13^C-NMR, DMSO-d_6_ (δ ppm): 155.82, 153.07, 151.18, 127.30, 115.18, 112.09. Anal. Calcd.: C, 36.37; H, 3.05. Found: C, 36.56; H, 3.07.

#### 3.1.3. Procedure for the Preparation of N^2^-alkylated Arylsemicarbazone **4a**

##### 5-Nitro-2-(N^2^-n-butyl)furfuraldehyde Semicarbazone (**4a**)

Compound **4a** was obtained following the literature procedure described by Brondani et al. [22]. M.F.: C_10_H_14_N_4_O_4_, M.W.: 254.24, orange solid; yield: 65%; m.p.: 110–112 °C; Rf: 0.66 (ethyl acetate/hexane 6:4). ^1^H-NMR: CDCl_3_ (δ ppm): 0.97 (t, 3H, CH_3_, *J* = 7.2 Hz); 1.35–1.45 (m, 2H, CH_2_); 1.52–1.60 (m, 2H, CH_2_); 3.95 (t, 2H, CH_2_N, *J* = 7.6 Hz); 5.01 (s, 1H, NH_2_); 6.52 (s, 1H, NH_2_); 6.81 (d, 1H, *J* = 3.6 Hz, Ar-H3); 7.39 (d, 1H, *J* = 3.6 Hz, Ar-H4); 7.43 (s, 1H, CH=N). ^13^C-NMR, CDCl_3_ (δ ppm): 155.92, 152.75, 151.79, 123.54, 113.45, 110.65, 40.77, 27.21, 20.05, 13.71. Anal. Calcd.: C, 47.24; H, 5.55. Found: C, 47.25; H, 5.62.

### 3.2. Molecular Modeling

To elucidate the binding mode of the compound **3c** that showed the highest activity among the compounds, docking calculations were done against CDK2, CDK5, CDK9, CLK1, DYRK1A, PIM1 and CK1δ. For comparison purposes, the compound **3g**, with IC_50_ > 10 μM, were also included, as a negative result. The compound **4a**, that showed activity against CK1δ target was also evaluated. The optimized structure for ligands **3c, 3g** and **4a** were obtained by application of the RM1 method [61], available as part of SPARTAN program [62], using internal default settings for convergence criteria. The targets structures used in the docking studies were taken from the RCSB Protein Data Bank, available online: http://www.rcsb.org (accessed on: 17 July 2019), under the following codes: CDK2 (6GUE), CDK5 (1UNL), CDK9 (3MY1), CLK1 (6FT8), DYRK1A (4YLK), PIM1 (3VBV) and CK1δ (3UYT). GOLD software [63] was used for these calculations. The binding mode for the ligands was determined as the highest (most positive) score among the possible solutions for each ligand, generated according to the Chemscore fitness function. The active site was defined after performing the alignment of all six targets, as all atoms within a radius of 7.0 Å from the co-crystallized ligand of CDK2 “FB8”. Ten aminoacids residues in each target were treated as flexible during the calculations, in order to take into account the effects of the induced fit, see Table S1 at Supplementary Materials. Then, BINANA program [64] was used to analyze the molecular interactions found in the docking results, using default settings, with the exception of the hydrogen bond distance that was changed to 3.5 Å.

### 3.3. Biological Activity

#### 3.3.1. Ethical Aspects of the Peripheral Blood Mononuclear Cell Assay

The project was submitted and approved by the Human Research Ethics Committee of the Federal University of Pernambuco, which is in accordance with Resolution 466/12 of the National Health Council and approved with CAAE opinion 67004017.3.0000.5208.

#### 3.3.2. Isolation of Peripheral Blood Mononuclear Cells

Mononuclear cells were obtained from the peripheral blood of healthy volunteers by heparin tube collection and isolated by Ficoll-Histopaque gradient centrifugation. After centrifugation, the cells were harvested, washed and resuspended in RPMI 1640 medium supplemented with 20% fetal bovine serum, 2 mM glutamine, 100 U/mL penicillin and 100 μg/mL streptomycin (Gibco™ Life Technologies, Waltham, MA, USA). The phytohemagglutinin mitogen 2% was also added into the culture medium. After that, using the neubauer chamber, the cells were counted and plated in a 96-well plate at a concentration of 10^6^ cells/mL and then were maintained for overnight under incubation at 37 °C and 5% CO_2_ [65].

#### 3.3.3. Culture of Tumor Cells

The cells were obtained from the Cell Bank of Rio de Janeiro and maintained in the Laboratory of Cell Culture of the Department of Antibiotics of the Federal University of Pernambuco. The lines K562 (myeloblastic leukaemia), HL-60 (promyelocytic leukemia) and MOLT-4 (lymphoblastic leukemia) were cultured in RPMI 1640 medium and the lines HEp-2 (larynx carcinoma), NCI-H292 (lung carcinoma), HT-29 (colon adenocarcinoma) and MCF-7 (breast adenocarcinoma) were cultured in DMEM medium, supplemented with 10% fetal bovine serum, 2 mM glutamine, 100 U/mL penicillin and 100 μg/mL streptomycin (Gibco™ Life Technologies), at 37 °C and 5% CO_2_.

#### 3.3.4. Cytotoxicity Assessment by MTT Assay

The evaluation of the cytotoxicity of the compounds was performed by the method of 3-(4,5-dimethyl-2-thiazolyl)-2,5-diphenyl-2*H*-tetrazolium bromide (MTT) (Sigma Aldrich Co., St. Louis, MO, USA). To perform the assay the cells were seeded in 96-well plates at the following concentrations: HL-60 and K562 (0.3 × 10^6^ cells/mL); MOLT-4 and PBMC (10^6^ cells/mL); NCI-H292, HT-29, HEp-2 e MCF-7 (10^5^ cells/mL). Briefly, the compounds previously dissolved in DMSO (dimethylsulfoxide, Vetec, São Paulo, Brazil), were diluted in medium and added to each well at the desired concentrations. For the calculation of the concentration that inhibits 50% of cell growth (IC_50_) the compounds were serially diluted to obtain the final concentrations between 0.19–25 μg/mL. Doxorubicin was used as the standard in the concentration range between (0.078–10 μg/mL). After 72 h of contact of the cells with the compounds, it was added to each well 25 μL of MTT solution (5 mg/mL). Plates were left for another 3 h in incubator at 37 °C and at the end of that period the supernatant was aspirated. To perform the reading, 100 μL of DMSO was added in each well for the dissolution of the formazan crystals. The amount of formazan was measured by reading the plate at 560 nm absorbance. The IC_50_ was calculated from the nonlinear regression in the Prisma 7.0 software (GraphPad, San Diego, CA, USA). Each sample was tested in triplicate in three independent experiments.

#### 3.3.5. Kinase Inhibition Assays

Kinase activities were assayed in appropriate kinase buffer, with either protein or peptide as substrate in the presence of 10 µM ATP in a final volume of 6 µL using the ADP-Glo^TM^ assay kit (Promega, Madison, WI, USA, as described in Zegzouti et al. [66]). Controls were performed with appropriate dilutions of dimethylsulfoxide (solvent of the tested compounds). Peptide substrates were obtained from Proteogenix (Schiltigheim, France).

The buffers used for kinase assays are the following: (A) 10 mM MgCl_2_, 1 mM EGTA, 1 mM DTT, 25 mM Tris-HCl pH 7.5, 50 µg/mL heparin; (B) 25 mM MOPS, pH 7.2, 12.5 mM -glycerolphosphate, 25 mM MgCl_2_, 5 mM EGTA, 2 mM EDTA, 0.25 mM DTT.

A panel of ten native or recombinant protein kinases was used during this study: 

*Hs*PIM1 (human proto-oncogene, recombinant, expressed in bacteria) was assayed in buffer B with 0.8 µg/µL of histone H1 (Sigma #H5505) as substrate.

*Hs*HASPIN (human, kinase domain, amino acids 470 to 798, recombinant, expressed in bacteria) was assayed in buffer A with 0.007 µg/µL of Histone H3 (1-21) peptide (ARTKQTARKSTGGKAPRKQLA) as substrate;

*Hs*CDK2/CyclinA (human, cyclin-dependent kinase-2, kindly provided by Dr. A. Echalier-Glazer, Leicester, UK) was assayed in buffer A with 0.8 µg/µL of histone H1 as substrate.

*Hs*CDK9/CyclinT (human, recombinant, expressed by baculovirus in Sf9 insect cells) was assayed in buffer A with 0.27 µg/µL of the following peptide: YSPTSPSYSPTSPSYSPTSPSKKKK, as substrate.

*Hs*CDK5/p25 (human, recombinant, expressed in bacteria) was assayed in buffer A, with 0.8 µg/µL of histone H1 as substrate.

HsAURKB (Human Aurora kinase B, recombinant, expressed by baculovirus in Sf9 insect cells, SignalChem, product #A31-10G) was assayed in buffer B with 0.2 µg/µL of MBP as substrate.

*Ssc*GSK-3α/β (*Sus scrofa domesticus*, glycogen synthase kinase-3, affinity purified from porcine brain) was assayed in buffer A with 0.010 µg/µL of GS-1 peptide, a GSK-3-selective substrate (YRRAAVPPSPSLSRHSSPHQSpEDEEE, “Sp” stands for phosphorylated serine).

SscCK1δ/ε (*Sus scrofa domesticus*, casein kinase 1δ/ε, affinity purified from porcine brain) was assayed in buffer A, with 0.022 μg/μL of the following peptide: RRKHAAIGSpAYSITA (“Sp” stands for phosphorylated serine) as CK1-specific substrate.

*Rn*DYRK1A (*Rattus norvegicus*, amino acids 1 to 499 including the kinase domain, recombinant, expressed in bacteria, DNA vector kindly provided by Dr. W. Becker, Aachen, Germany) was assayed in buffer A with 0.033 μg/µL of the following peptide: KKISGRLSPIMTEQ as substrate.

*Mm*CLK1 (from *Mus musculus*, recombinant, expressed in bacteria) was assayed in buffer A with 0.027μg/µL of the following peptide: GRSRSRSRSRSR as substrate.

#### 3.3.6. Flow Cytometry Analysis

HL-60 cells (3 × 10^5^) were plated in 24-well plates and treated with the compounds **3c** (13 and 26 μM) and **4a** (11 and 22 μM) for 72 h at 37 °C with 5% CO_2_. In all trials, doxorubicin was used as a positive control. After the incubation period, an aliquot of 500 μL of treated and untreated cells suspension was transferred to an Eppendorf tube and centrifuged to remove the supernatant and continue the protocol of the test. In the cell viability and annexin V assays, kits were used, ViaCount kit and guava nexin kit (Guava Technologies), respectively. The protocols performed in these tests followed the manufacturer’s instructions. For to analyze mitochondrial depolarization, rhodamine 123 fluorescent dye was used. In the assay for cell cycle analysis and DNA fragmentation propidium iodide was used. The experiments were performed on Guava EasyCyte HT (Merck-Millipore) flow cytometer, acquiring 5000 events. The data were analyzed in Guava-Soft™ software version 2.7. Three independent experiments were performed in triplicate.

## 4. Conclusions

In this study, two arylsemicarbazone analogs were identified as valuable compounds for the development of novel anticancer agents. Especially, cytotoxic properties were directed against HL-60 (promyelocytic leukemia) cancer cell line associated with encouraging safety profile (SI PBMC = 6.2 and 4.5 for **3c** and **4a**, respectively). The proposed mechanism of action of **3c** was the induction of cell death through apoptosis by alteration in the mitochondria transmembrane potential. Regarding compound **4a**, DNA fragmentation in HL-60 cells was found to induce cytotoxic properties.

## Figures and Tables

**Table 1:** IC_50_ values (μM) of the arylsemicarbazone derivatives in tumor cell lines or Peripheral Blood Mononuclear Cells (PBMCs) performed by the MTT test after 72 h of incubation.

IC_50_ (μM)								
Compound	K562	HL-60	MOLT-4	HEp-2	NCI-H292	HT-29	MCF-7	PBMC
**3a**	> 100	> 100	> 100	> 100	> 100	> 100	> 100	> 100
**3b**	> 100	> 100	> 100	> 100	> 100	> 100	> 100	> 100
**3c**	52.34 ± 1.15	13.08 ± 0.62	41.00 ± 0.7	37.08 ± 1.50	56.27 ± 3.05	40.57 ± 1.02	34.02 ± 1.06	82.01 ± 1.63
**3d**	25.19 ± 2.43	16.93 ± 1.10	37.17 ± 0.84	23.13 ± 0.80	> 100	> 100	33.46 ± 0.75	46.26 ± 0.97
**3e**	58.69 ± 0.97	> 100	> 100	22.26 ± 0.40	> 100	> 100	33.90 ± 0.80	> 100
**3f**	26.28 ± 1.86	14.22 ± 1.41	> 100	8.61 ± 0.35	> 100	> 100	23.26 ± 1.55	> 100
**3g**	33.29 ± 0.71	48.53 ± 1.63	56.43 ± 0.97	24.26 ± 0.97	> 100	> 100	> 100	> 100
**3h**	> 100	> 100	25.53 ± 1.9	42.86 ± 0.75	48.79 ± 0.97	46.97 ± 0.75	58.37 ± 1.86	49.70 ± 1.06
**3i**	> 100	> 100	> 100	> 100	> 100	> 100	> 100	> 100
**3j**	> 100	> 100	> 100	> 100	> 100	> 100	> 100	> 100
**3k**	> 100	> 100	> 100	> 100	> 100	> 100	> 100	> 100
**3l**	> 100	> 100	> 100	> 100	> 100	> 100	> 100	> 100
**3m**	N.T.	24.33 ±0.16	N.T.	N.T.	31.9±0.07	28.42± 8.56	8.56 ±0.01	>100
**4a**	24.21 ± 1.15	11.38 ± 0.27	19.53 ± 0.62	34.37 ± 0.88	37.89 ± 1.50	36.32 ± 0.97	33.59 ± 0.71	51.21 ± 0.75
Doxo ^a^	1.47 ± 0.09	0.11 ± 0.01	0.73 ± 0.08	1.28 ± 0.11	0.18 ± 0.02	0.73 ± 0.09	0.20 ± 0.03	5.33 ± 0.62

Data are expressed as mean ± SD from three different experiments. N.T.: Not tested. ^a^ Doxo (doxorubicin) was used as a positive control.

**Table 2:** Physicochemical parameters of compounds **3a-m** and **4a**: ClogD_7.4_ and aqueous solubility.

Compound	Structure	ClogD_7.4_	LogS	Solubility (mg/mL)
**3a**	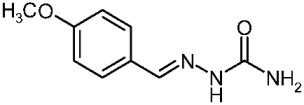	0.99	−2.27	1.038
**3b**	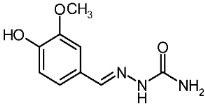	0.70	−1.87	2.822
**3c**	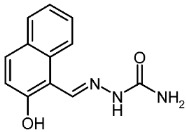	1.93	−3.72	0.044
**3d**	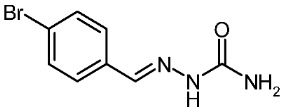	2.03	−3.36	0.106
**3e**	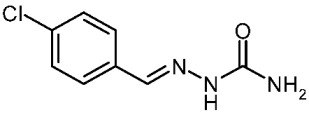	1.76	−3.01	0.193
**3f**	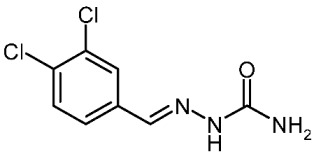	2.28	−3.76	0.040
**3g**	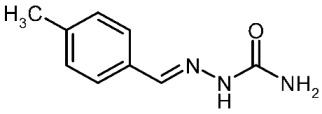	1.71	−2.76	0.308
**3h**	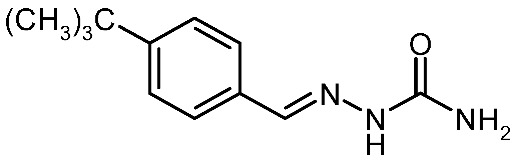	2.87	−3.87	0.030
**3i**	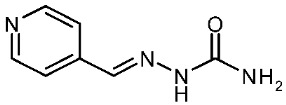	−0.07	−1.10	13.040
**3j**	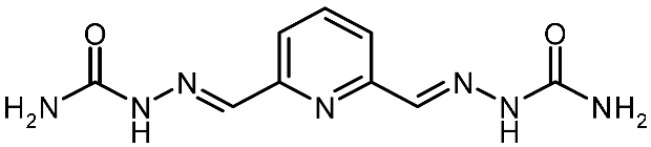	0.64	−2.46	0.864
**3k**	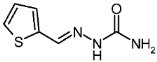	1.06	−2.24	0.974
**3l**	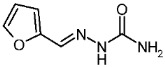	0.19	−1.62	3.674
**3m**	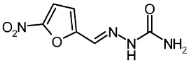	0.09	−2.90	0.249
**4a**	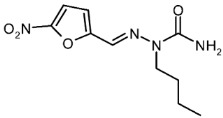	1.54	−4.24	0.015

ClogD_7.4_ and solubility at pH 7.4 were predicted using MarvinSketch v. 15.3.16. Solubility values at pH 7.4 can be classified in three categories: low solubility if s < 0.01 mg/mL, moderate solubility if 0.01 < s < 0.06 mg/mL and high solubility if s > 0.06 mg/mL.

**Table 3:** Kinase inhibitory activity of arylsemicarbazone derivatives.

Protein Kinases	IC_50_ (μM)							
	3c	3d	3e	3f	3g	3h	3m	4a
CDK2/CyclinA	**0.53**	> 10	> 10	> 10	> 10	> 10	N.T.	> 10
CDK5/p25	**0.35**	> 10	> 10	> 10	> 10	> 10	> 10	> 10
CDK9/CyclinT	**0.86**	> 10	> 10	> 10	> 10	> 10	> 10	> 10
HASPIN	> 10	> 10	> 10	> 10	> 10	> 10	> 10	> 10
PIM1	**0.52**	> 10	> 10	> 10	> 10	> 10	> 10	> 10
CLK1	**0.29**	> 10	> 10	> 10	> 10	**1.9**	N.T.	> 10
DYRK1A	**0.073**	> 10	> 10	> 10	> 10	> 10	N.T.	> 10
CK1δ/ε	> 10	> 10	> 10	> 10	> 10	> 10	N.T.	**0.76**
GSK3 α/β	> 10	> 10	> 10	> 10	> 10	> 10	> 10	> 10
AURKB	**5.1**	> 10	> 10	> 10	> 10	> 10	N.T.	> 10

Data expressed as mean ± SD of at least three assays. NT: no tested.

**Table 4:** Molecular interaction of CDK2 and DYRK1A with molecules **3c** and **3g**, and CK1δ with molecule **4a**.

CDK2			DYRK1A			CK1δ	
Residues	Molecules		Residues	Molecules		Residues	Molecule
	3c	3g		3c	3g		4a
ILE-10	3.1/3.1	2.8	ILE-165	2.9	2.9	ILE-15	HC
VAL-18	HC	-	ALA-186	HC	-	LYS-38	HC
ALA-31	HC	-	LYS-188	HC	HC	MET-82	HC
LYS-33	HC	HC	VAL-222	HC	HC	GLU-83	2.9
VAL-64	HC	HC	PHE-238	HC	HC	LEU-85	2.8
PHE-80	HC	HC	GLU-239	3.0	-	SER-88	2.9
ASP-86	2.9	2.9/3.0	LEU-241	2.9/HC	HC	LEU-135	HC
LEU-134	HC	HC	SER-242	3.0	3.1	ILE-148	HC
ALA-144	-	HC	LEU-294	HC	HC		
ASP-145	3.0	-	VAL-306	HC	HC		
Docking score	33.37	29.63	Docking score	35.00	28.64	Docking score	23.47

HC means hydrophobic contacts and the numbers are the hydrogen bond distances between donor and acceptor, in Ångströms.

## Supplementary Materials

The following materials are available online at https://www.mdpi.com/1424-8247/12/4/169/s1. ^1^H- and ^13^C-NMR spectra for compounds **3a-3m** and **4a**, as well as molecular docking calculation parameters
