# Supplementary material for: Biological Evaluation of Arylsemicarbazone Derivatives as Potential Anticancer Agents
Source: Pharmaceuticals (Basel). 2019 Nov 17;12(4):169. doi: 10.3390/ph12040169 (PMC6958387; doi:10.3390/ph12040169)
Supplement: Supplementary file 1 [file pharmaceuticals-12-00169-s001.pdf]

pharmaceuticals

Supplementary Materials

Biological Evaluation of Arylsemicarbazone Derivatives as Potential Anticancer Agents

Anne Cecília Nascimento da Cruz [1], Dalci José Brondani [2], Temístocles I´talo de Santana [1], Lucas Oliveira da Silva [2], Elizabeth Fernanda da Oliveira Borba [1], Antônio Rodolfo de Faria [2], Julianna Ferreira Cavalcanti de Albuquerque [1], Sylvie Piessard [3], Rafael Matos Ximenes [1], Blandine Baratte [4,5], Stéphane Bach [4,5], Sandrine Ruchaud [4], Francisco Jaime Bezerra Mendonça Junior [6], Marc-Antoine Bazin [3], Marcelo Montenegro Rabello [2], Marcelo Zaldini Hernandes [2], Pascal Marchand [3,*] and Teresinha Gonçalves da Silva [1,*]

1. Departamento de Antibióticos, Centro de Biociências, Universidade Federal de Pernambuco, Recife, PE, 50740-520, Brazil; annececilia2006@hotmail.com (A.C.N.C.); temistoclesitalo@gmail.com (T.I.S.); elizabethfernanda_7@hotmail.com (E.F.O.B.); julianna@ufpe.br (J.F.C.A.); ximenesrm@gmail.com (R.M.X.)
2. Departamento de Ciências Farmacêuticas, Centro de Ciências da Saúde, Universidade Federal de Pernambuco, Recife, PE, 50740-520, Brazil; brondani.dj@gmail.com (D.J.B); luc.osilva@gmail.com (L.O.S.); rodolfo.ufpe@gmail.com (A.R.F.); montenegro.rabello@gmail.com (M.M.R.); zaldini@gmail.com (M.Z.H.)
3. Université de Nantes, Cibles et Médicaments des Infections et du Cancer, IICiMed, EA 1155, Nantes, F-44000, France; sylvie.piessard@univ-nantes.fr (S.P.); marc-antoine.bazin@univ-nantes.fr (M.A.B.)
4. Sorbonne Université, CNRS, USR3151, « Protein phosphorylation and human diseases » Unit, Station Biologique, Roscoff, F-29688, France; baratte@sb-roscoff.fr (B.B.); bach@sb-roscoff.fr (S.B.); sandrine.ruchaud@sb-roscoff.fr (S.R.)
5. Sorbonne Université, CNRS, FR2424, Kinase Inhibitor Specialized Screening Facility - KISSf, Station Biologique, Roscoff, F-29688, France
6. Laboratory of Synthesis and Drug Delivery, Department of Biological Sciences, State University of Paraiba, João Pessoa, PB, 58071-160, Brazil; franciscojbmendonca@yahoo.com.br

* Correspondence: teresinha.goncalves@pq.cnpq.br (T.G.S.); pascal.marchand@univ-nantes.fr (P.M.); Tel.: +55 31 81 2126 8347 (T.G.S.); Tel.: +33 253 009 155 (P.M.)

1. 1H- & 13C-NMR Spectra for Compounds 3a-3m and 4a

Figure S1. 1H NMR at 400 MHz and 13C NMR at 100 MHz spectra, DMSO-d6, for compounds **3a.**

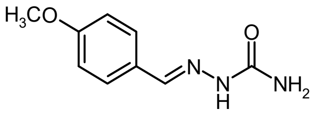

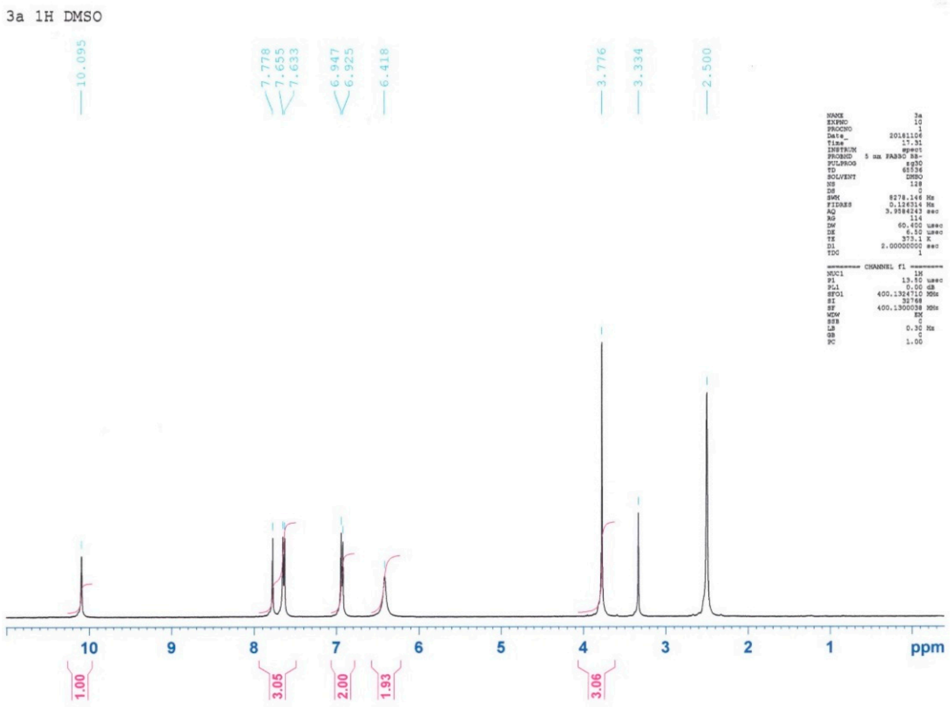

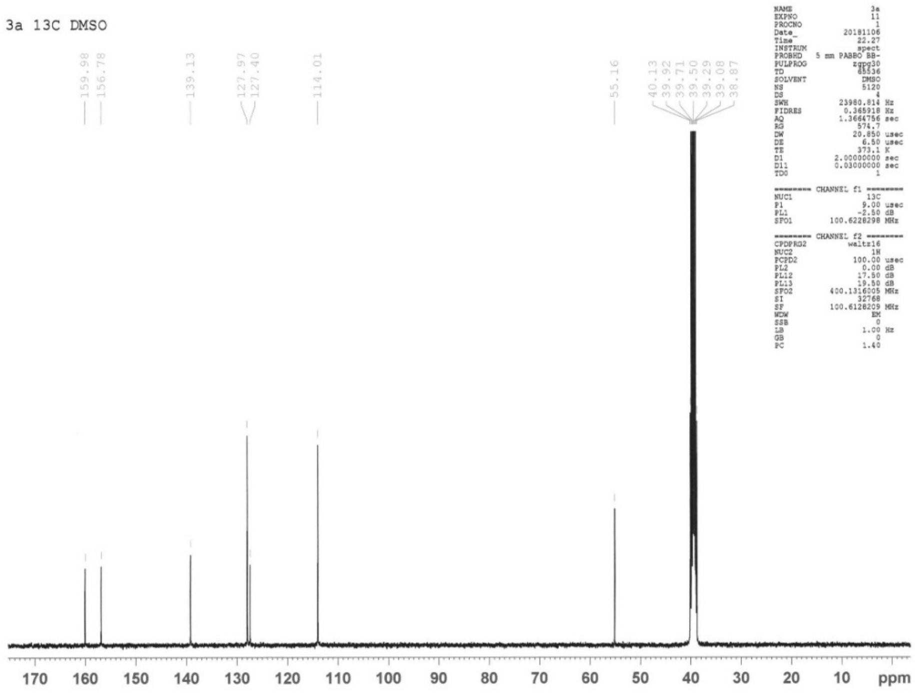

Figure S2. 1H NMR at 400 MHz and 13C NMR at 100 MHz spectra, DMSO-d6, for compounds **3b.**

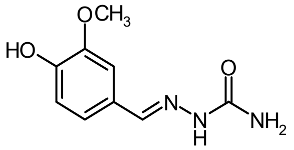

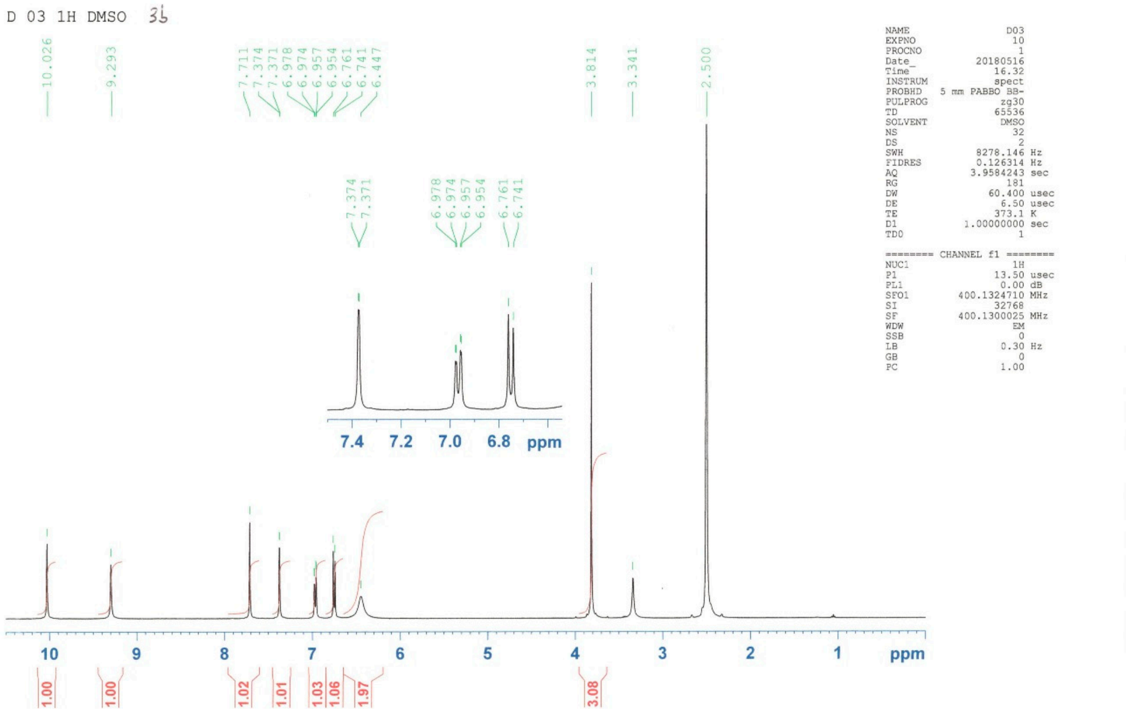

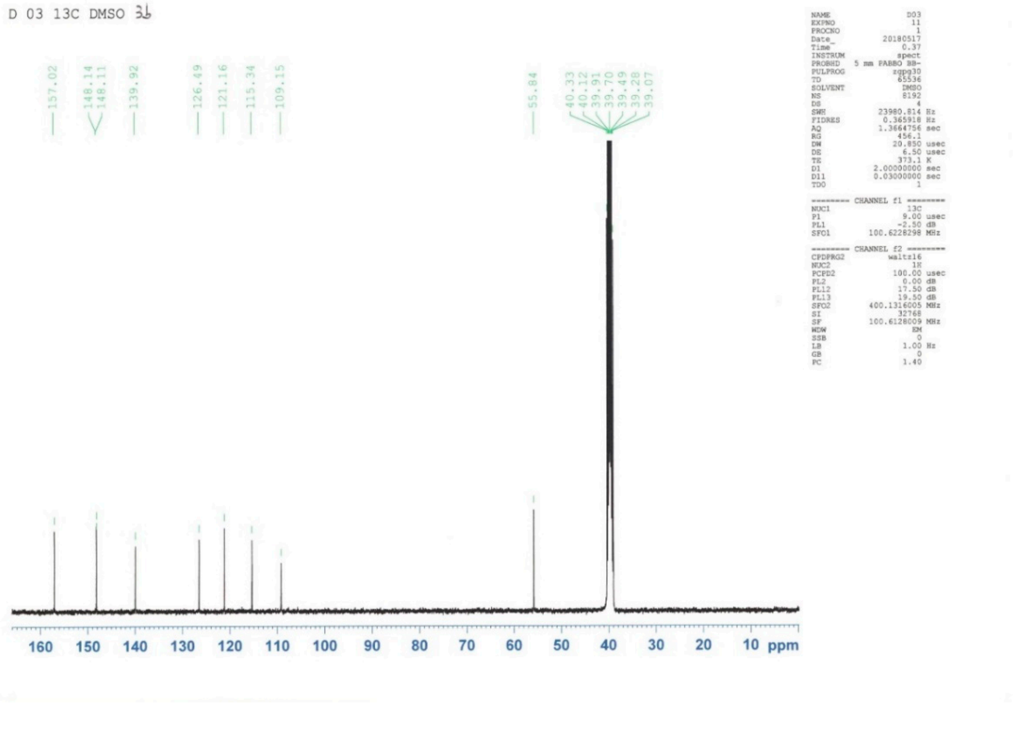

Figure S3. 1H NMR at 400 MHz and 13C NMR at 100 MHz spectra, DMSO-d6, for compounds **3c.**

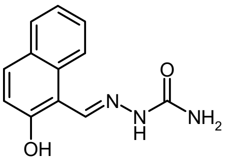

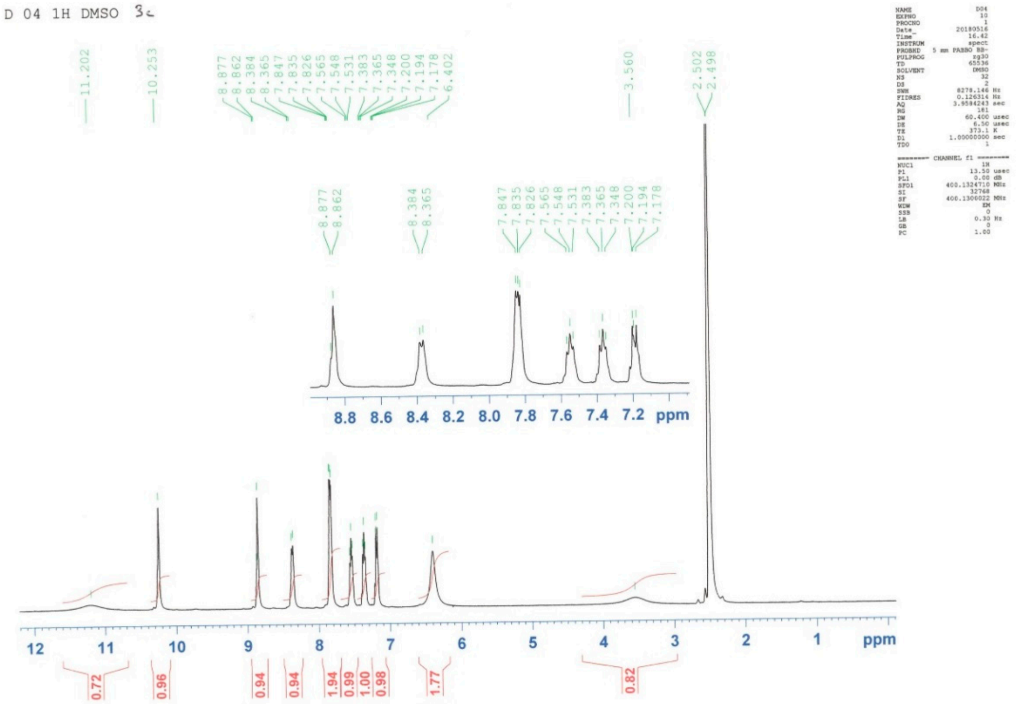

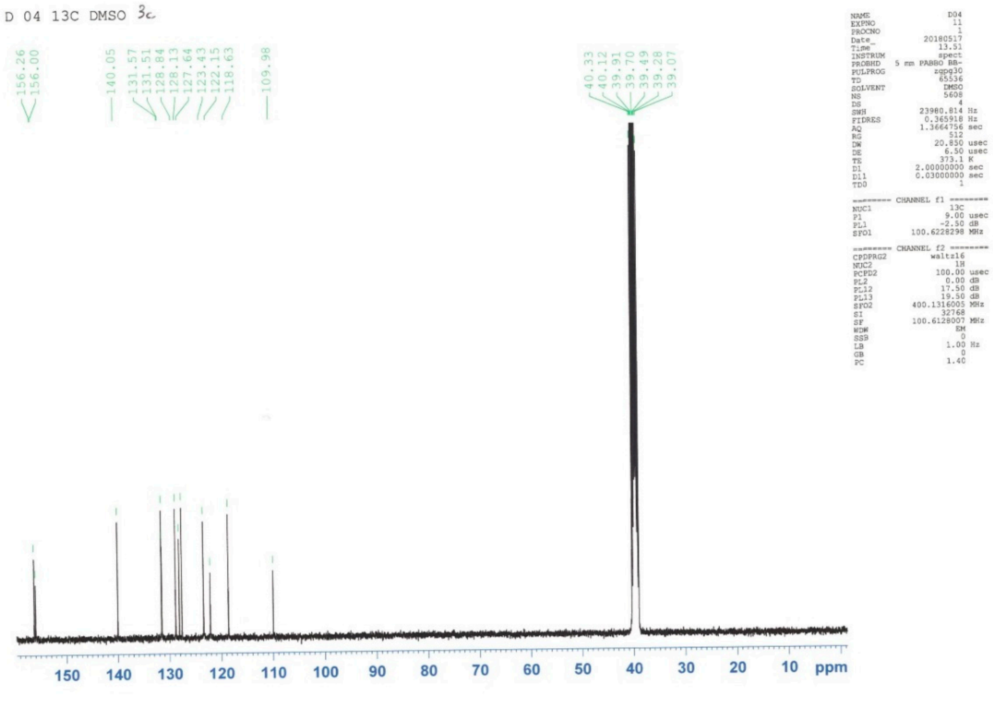

Figure S4. 1H NMR at 400 MHz and 13C NMR at 100 MHz spectra, DMSO-d6, for compounds **3d.**

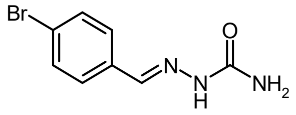

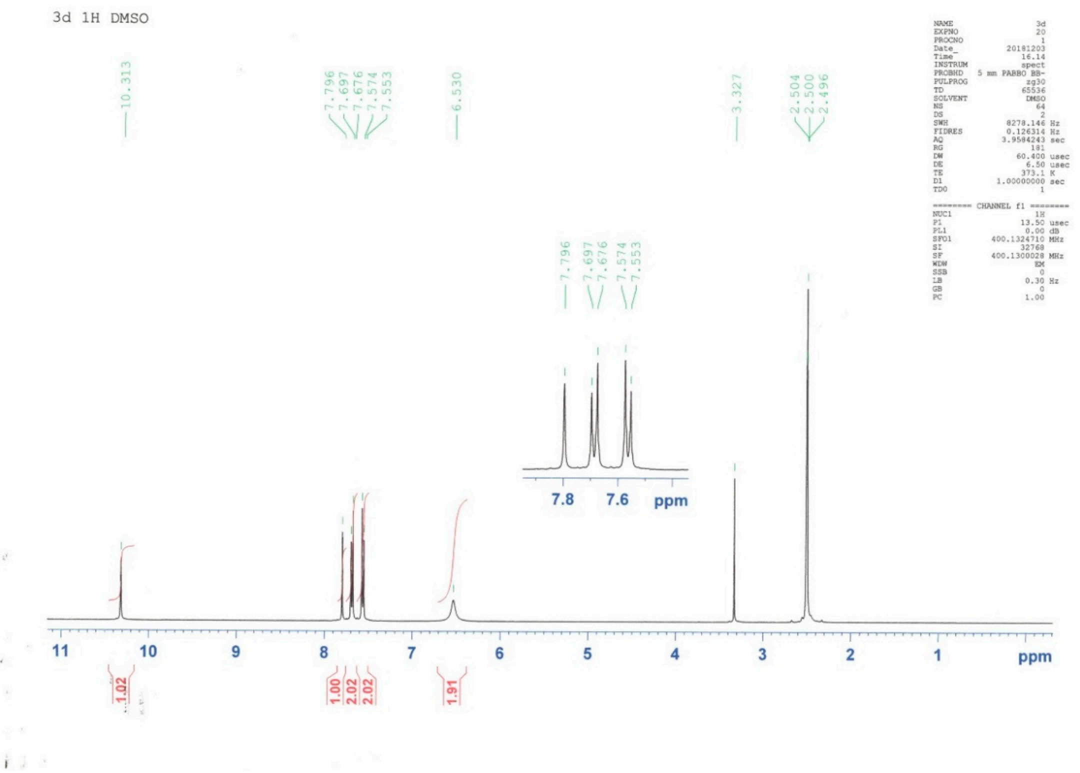

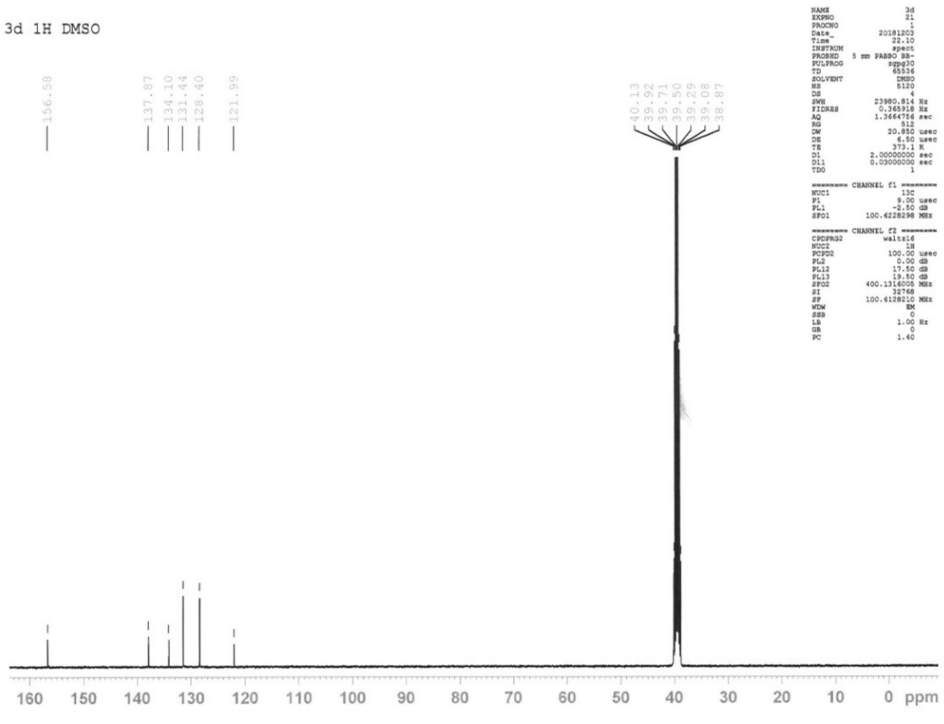

Figure S5. 1H NMR at 400 MHz and 13C NMR at 100 MHz spectra, DMSO-d6, for compounds **3e.**

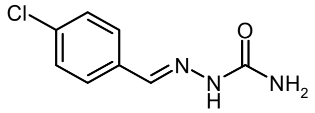

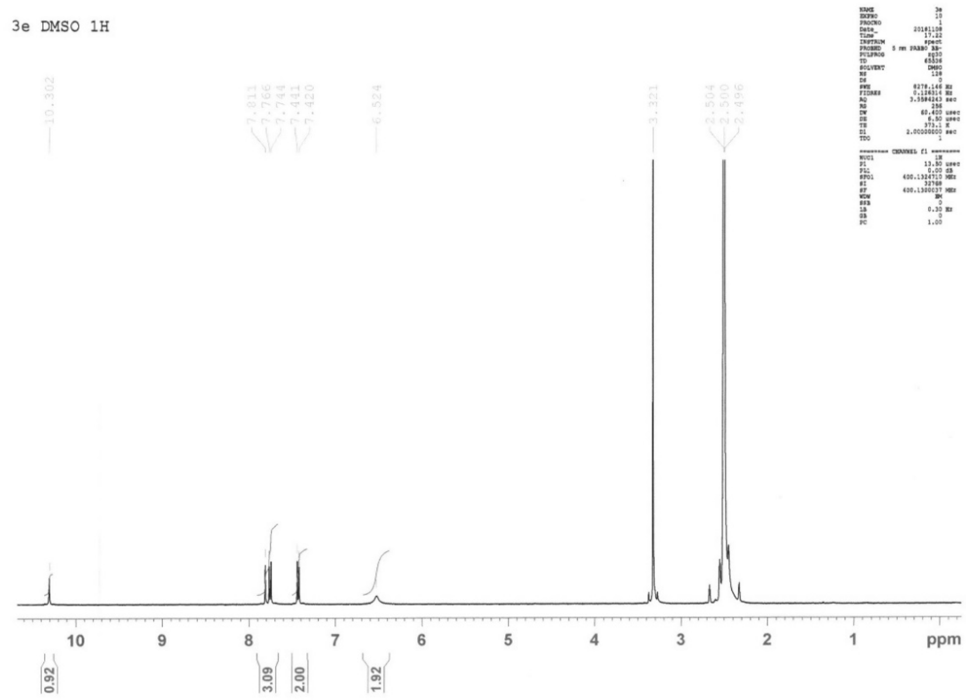

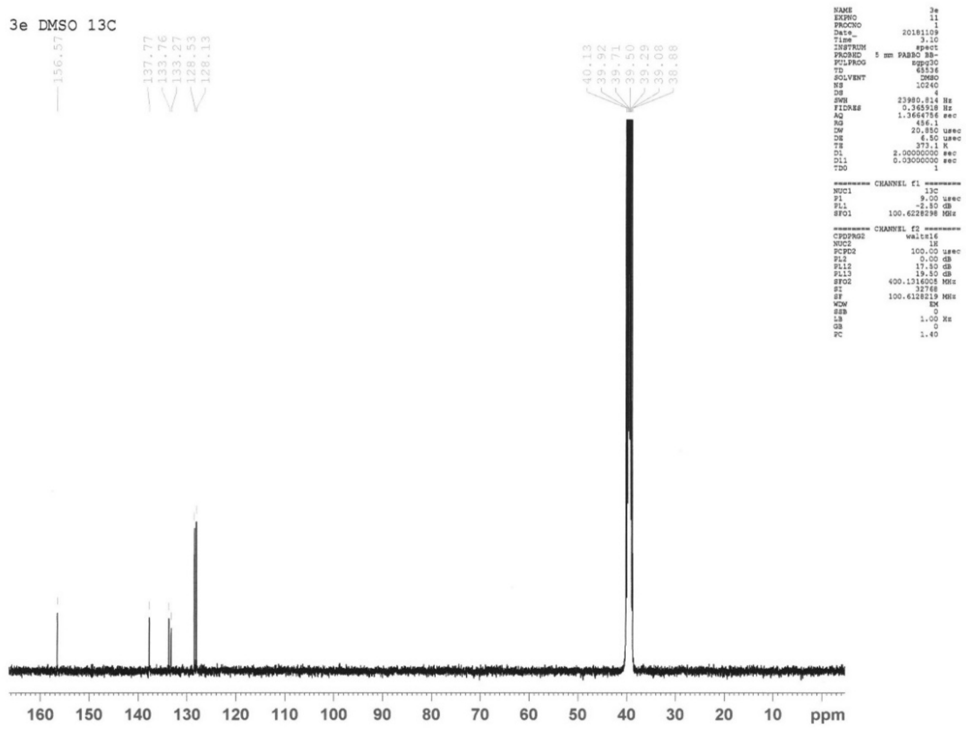

Figure S6. 1H NMR at 400 MHz and 13C NMR at 100 MHz spectra, DMSO-d6, for compounds **3f.**

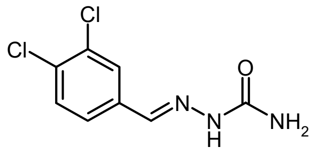

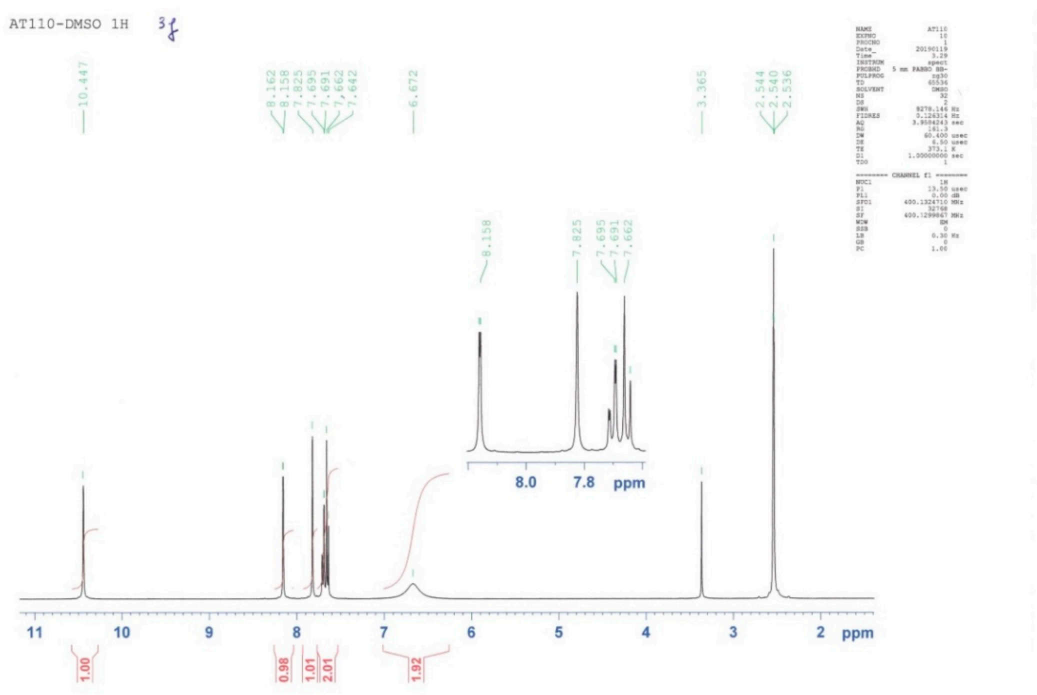

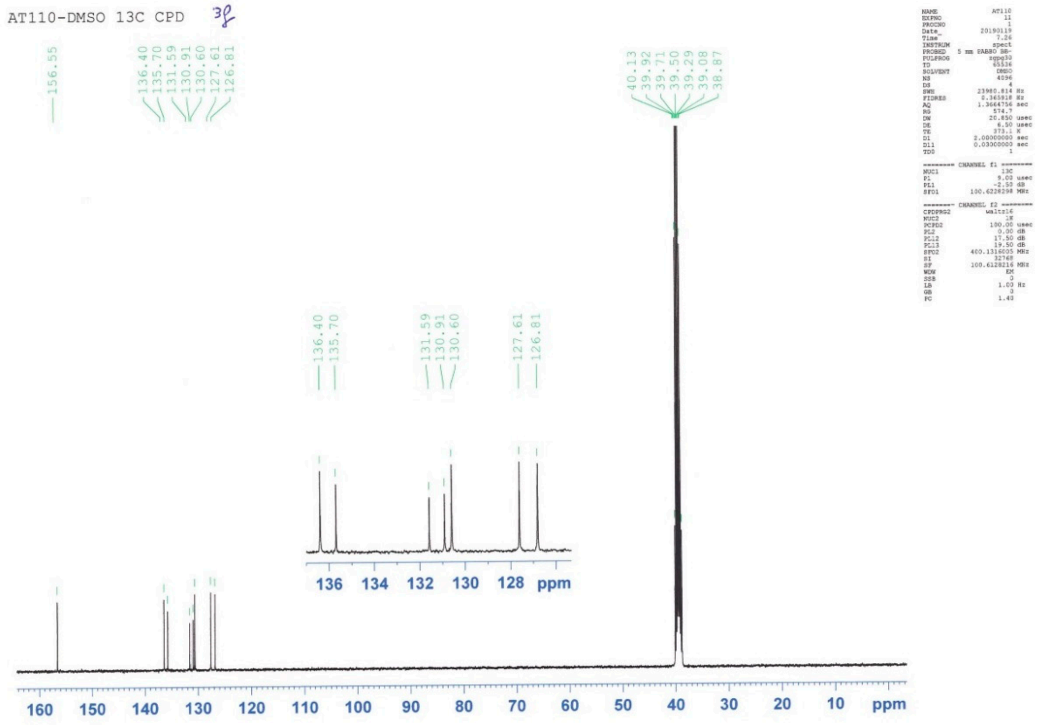

Figure S7. 1H NMR at 400 MHz and 13C NMR at 100 MHz spectra, DMSO-d6, for compounds **3g.**

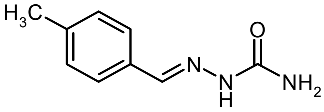

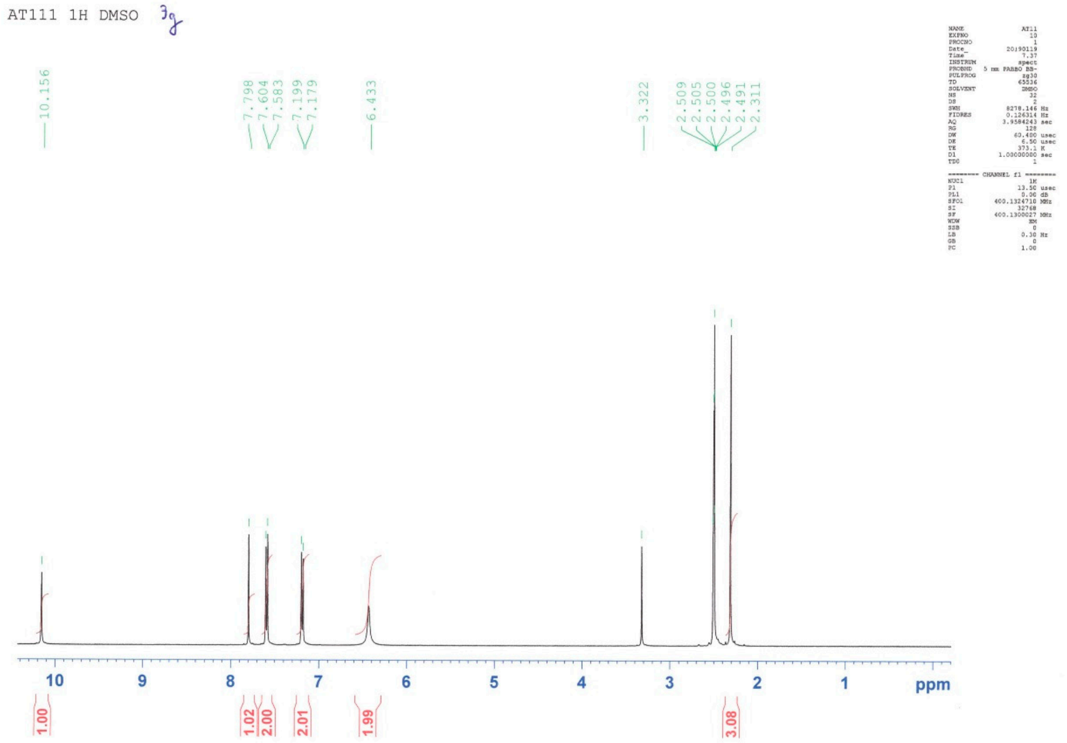

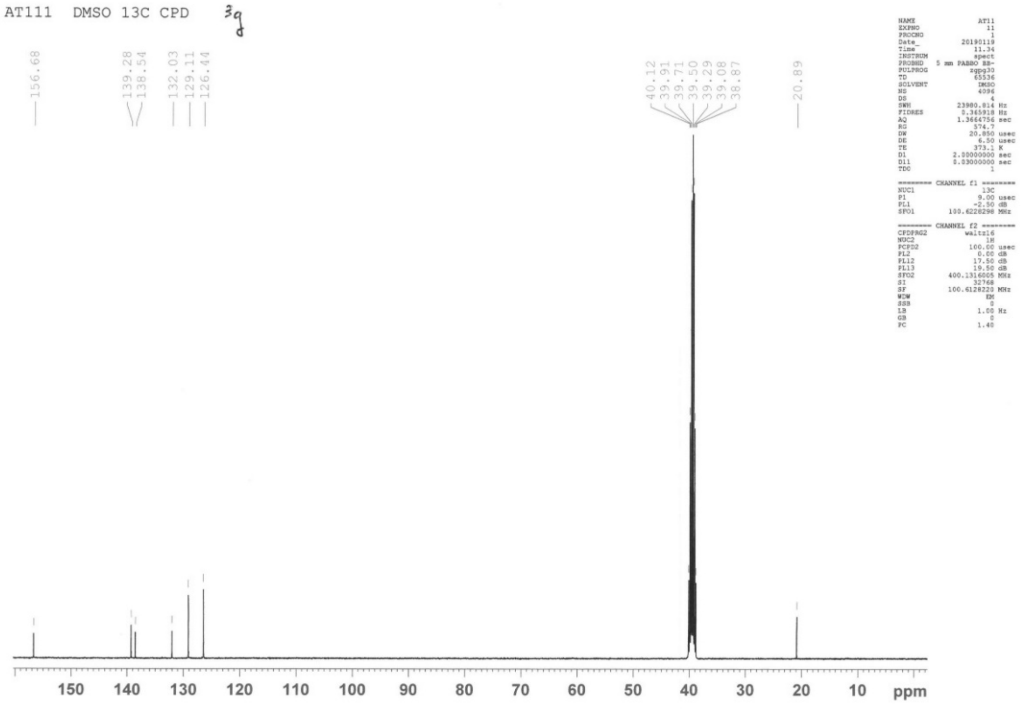

Figure S8. 1H NMR at 400 MHz and 13C NMR at 100 MHz spectra, DMSO-d6, for compounds **3h.**

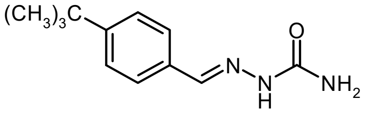

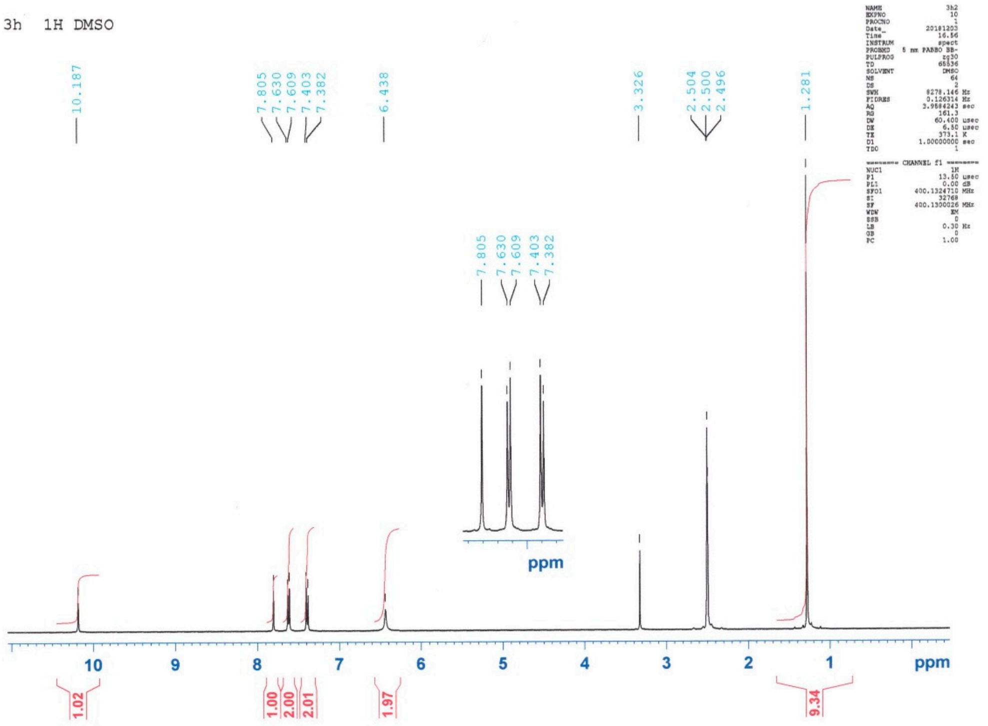

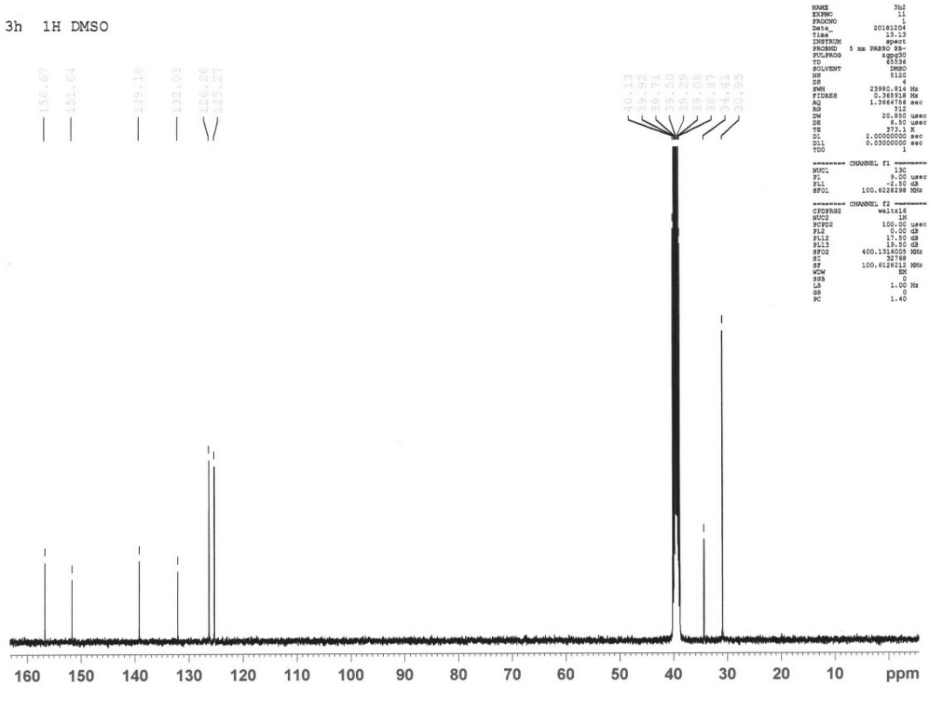

Figure S9. 1H NMR at 400 MHz and 13C NMR at 100 MHz spectra, DMSO-d6, for compounds **3i.**

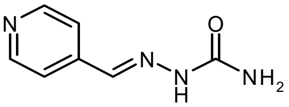

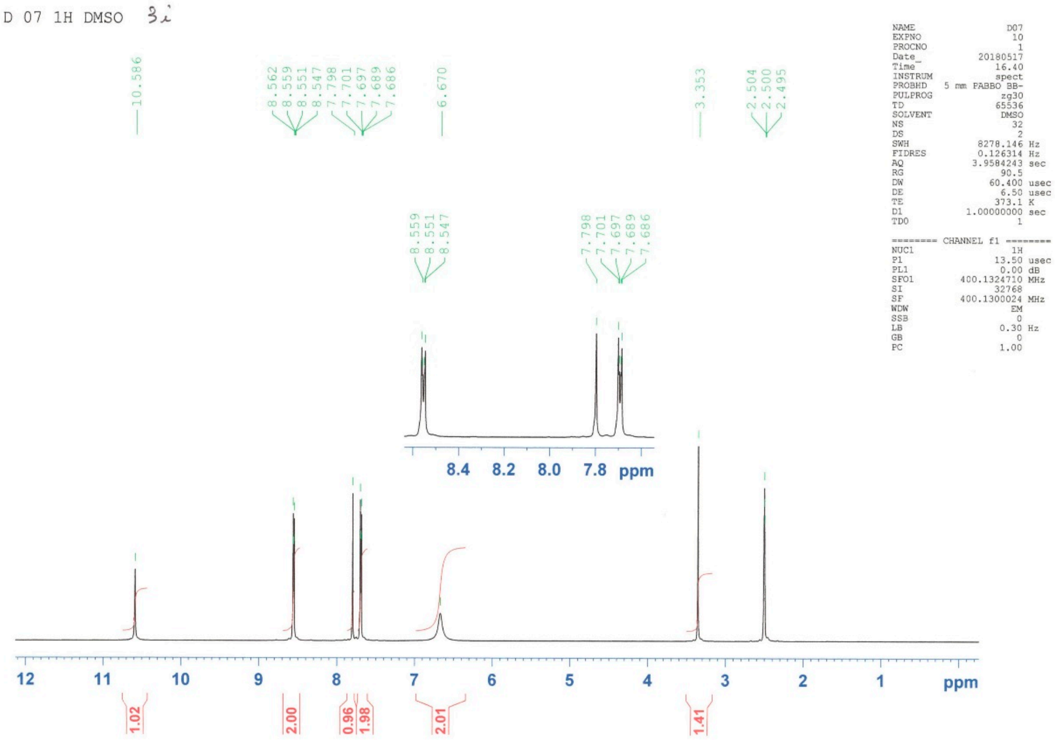

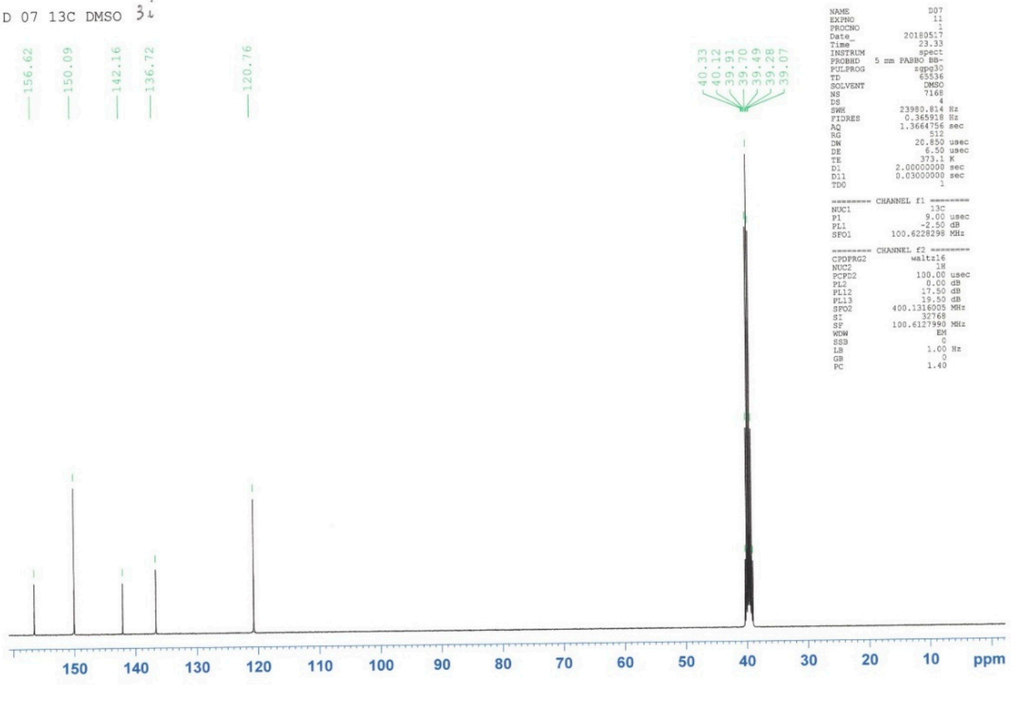

Figure S10. 1H NMR at 400 MHz and 13C NMR at 100 MHz spectra, DMSO-d6, for compounds **3j.**

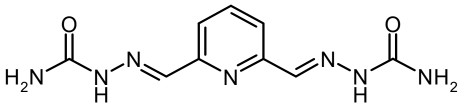

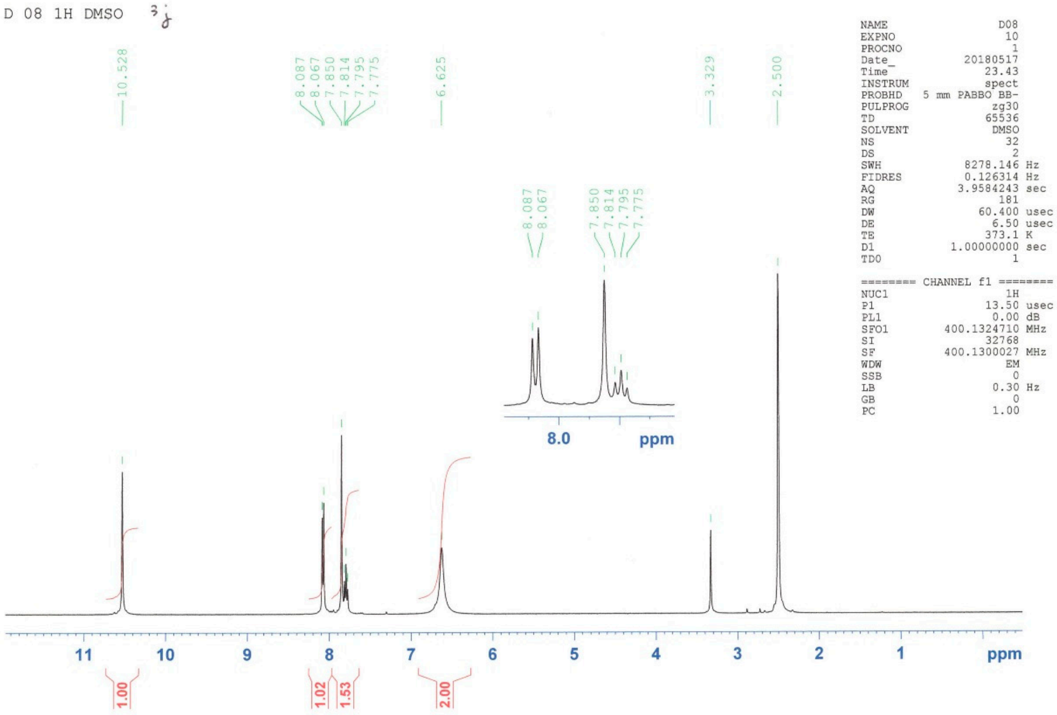

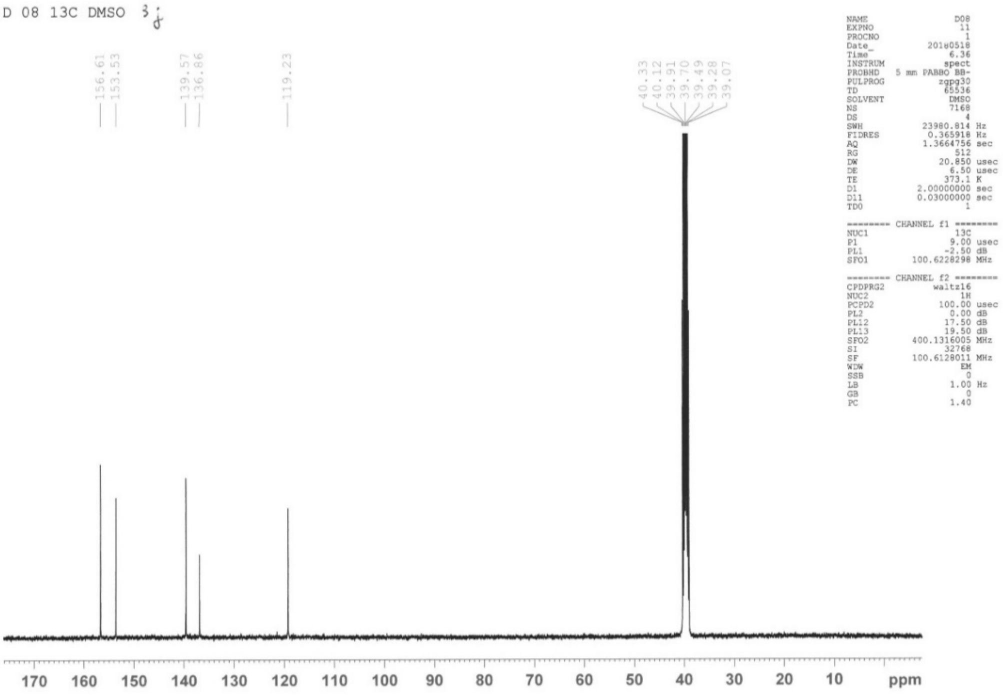

Figure S11. 1H NMR at 400 MHz and 13C NMR at 100 MHz spectra, DMSO-d6, for compounds **3k.**

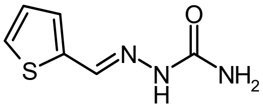

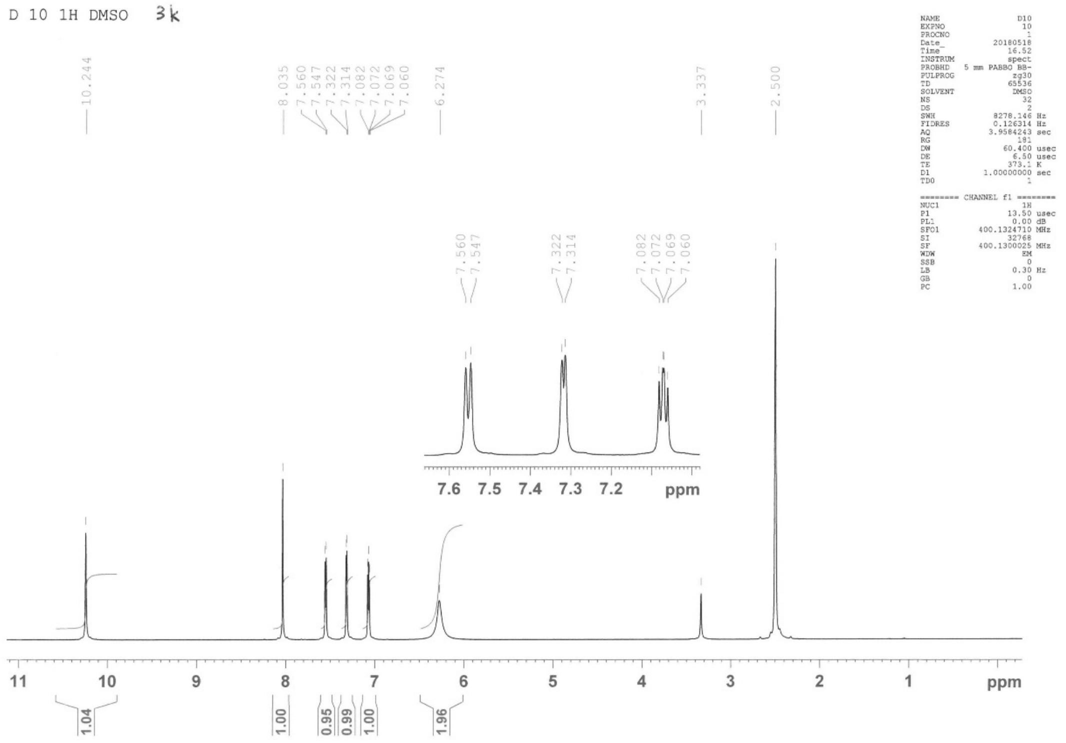

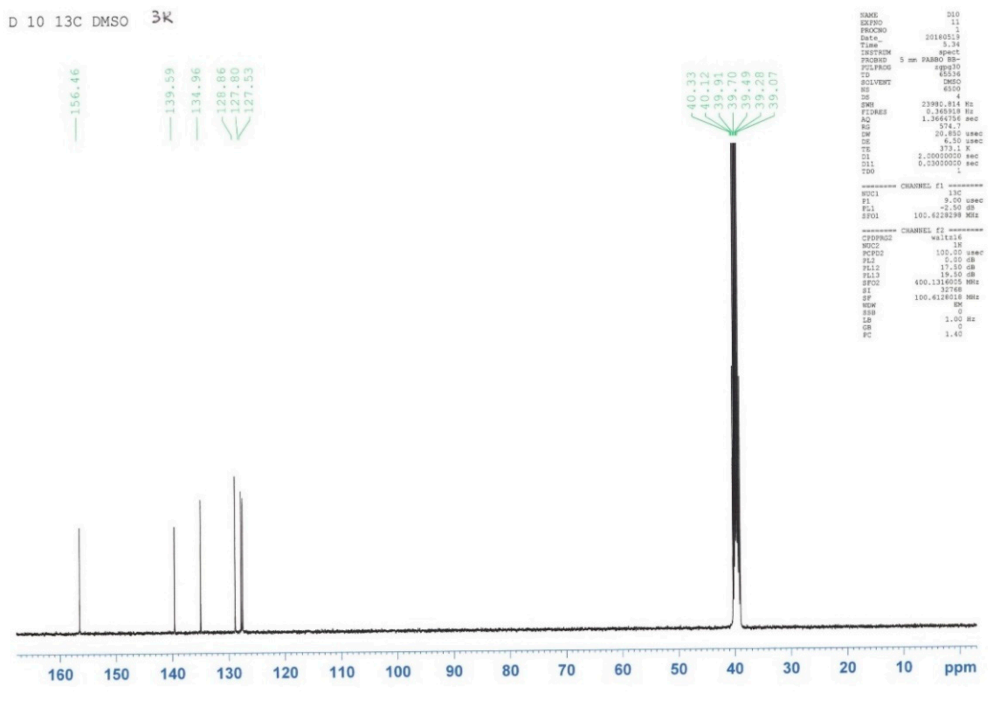

Figure S12. 1H NMR at 400 MHz and 13C NMR at 100 MHz spectra, DMSO-d6, for compounds **3l.**

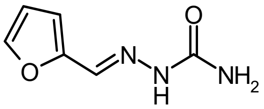

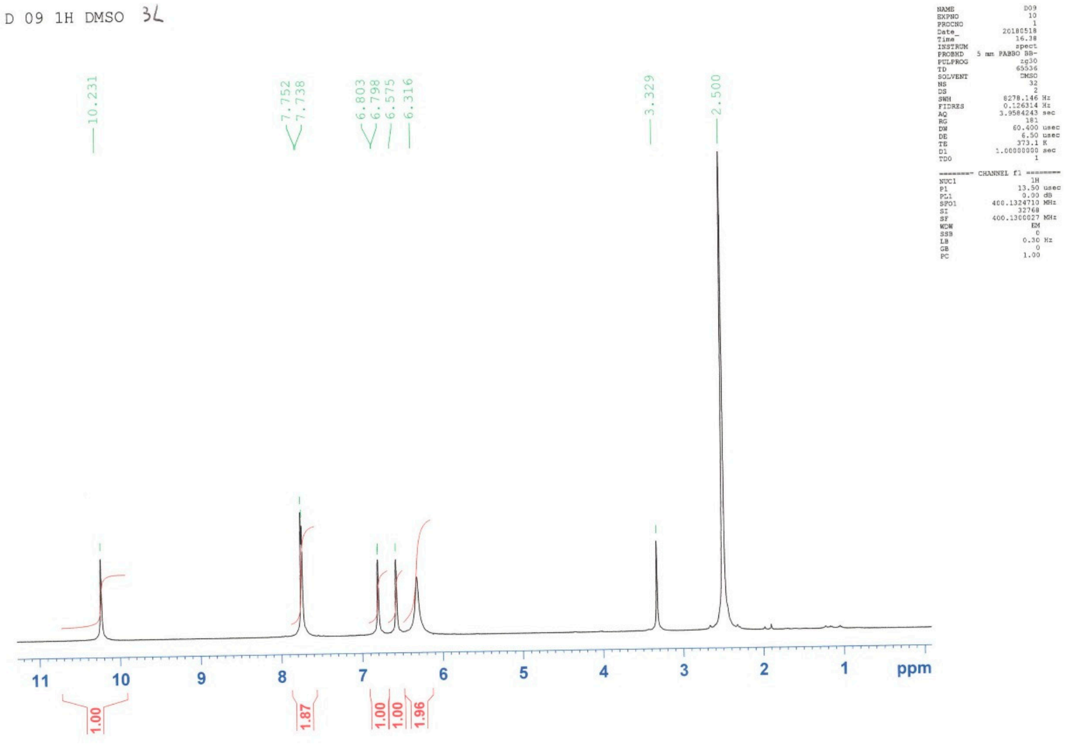

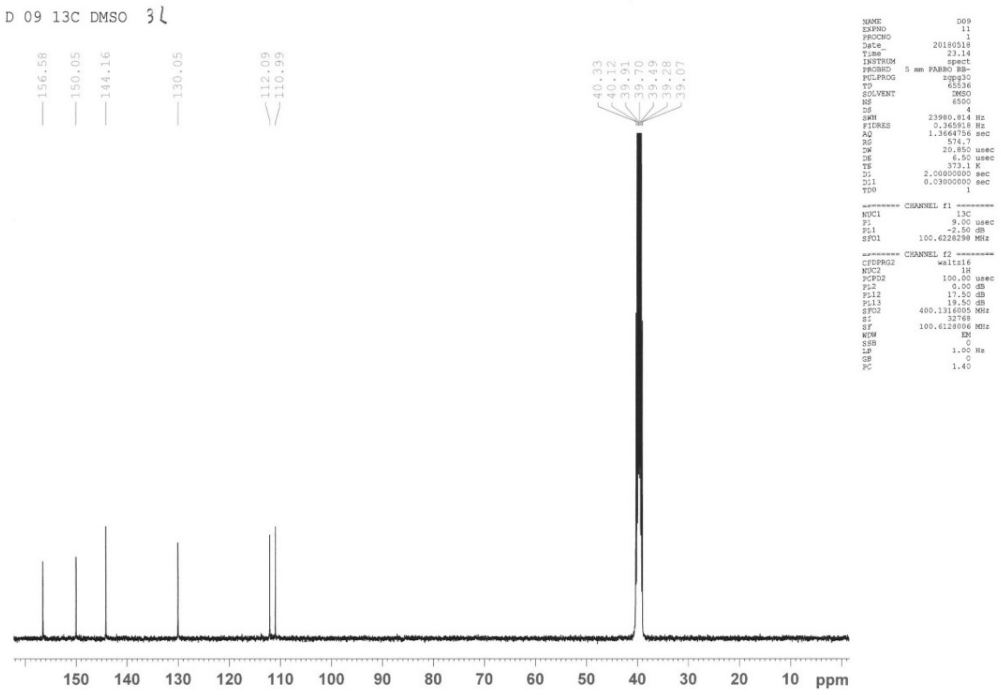

Figure S13. 1H NMR at 400 MHz and 13C NMR at 100 MHz spectra, DMSO-d6, for compounds **3m.**

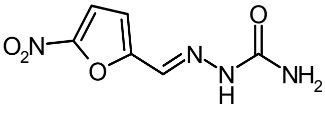

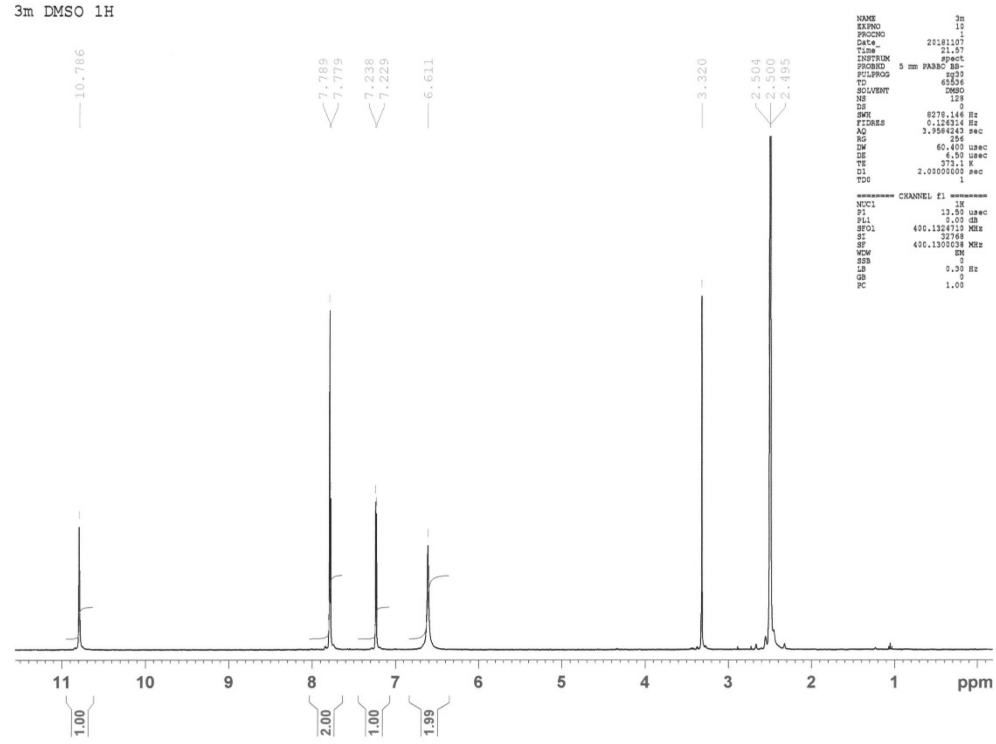

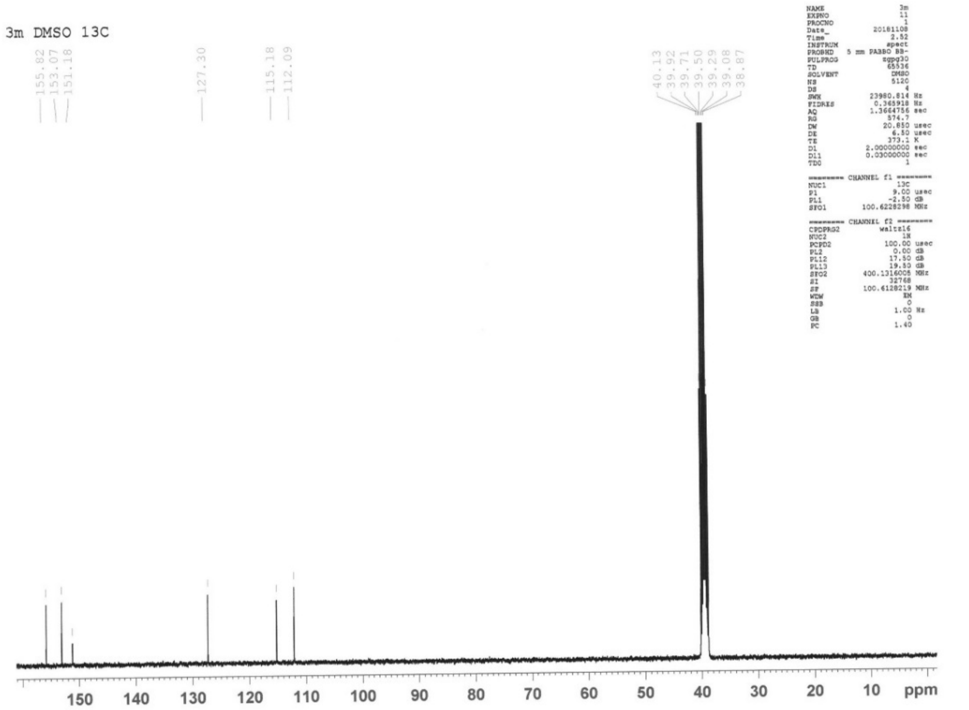

Figure S14. 1H NMR at 400 MHz and 13C NMR at 100 MHz spectra, CDCl3, for compounds **4a.**

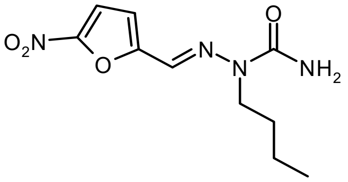

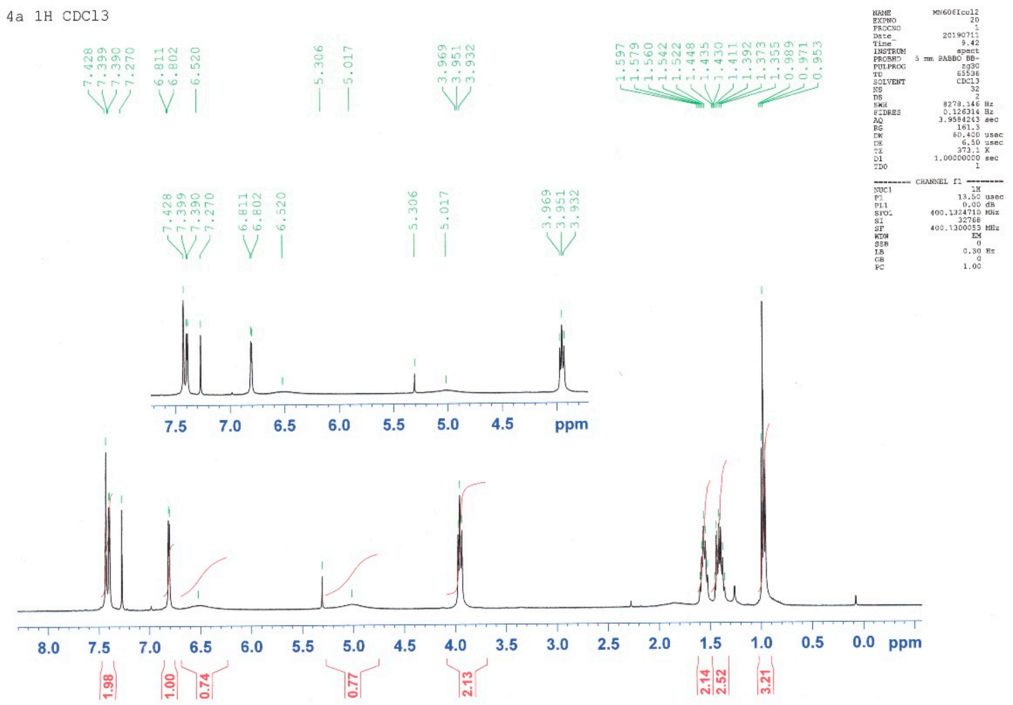

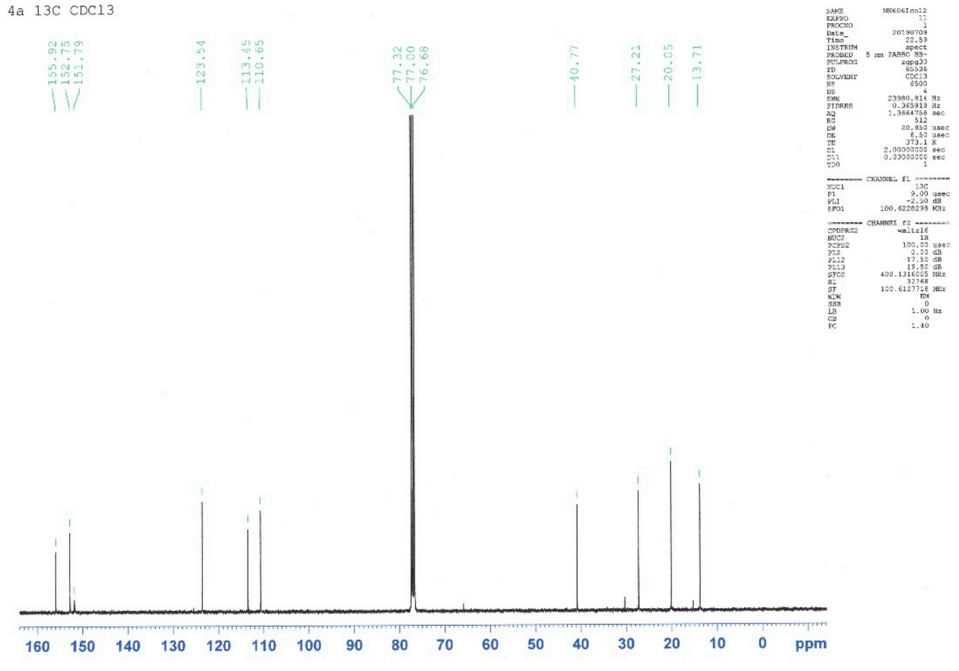

2. Molecular Docking Calculation Parameters

Table S1. Amino acid residues treated as flexible during the docking calculations.

CDK2	CDK5	CDK9	CLK1	DYRK1A	PIM1	CK1δ
LYS-33	LYS-33	GLN-27	PHE-172	PHE-170	PHE-49	ILE-15
GLU-51	GLU-51	LYS-48	LYS-191	LYS-188	LYS-67	LYS-38
PHE-80	PHE-80	PHE-103	GLU-206	GLU-203	LEU-120	MET-80
PHE-82	PHE-82	PHE-105	PHE-241	PHE-238	GLN-127	MET82
ASP-86	ASP-86	CYS-106	SER-247	ASN-244	ASP-128	LEU-84
LYS-89	LYS-89	GLU-107	ASP-250	ASP-247	ASP-131	ASP-91
GLN-131	GLN-130	ASP-109	GLU-292	GLU-291	GLU-171	ASP-132
ASN-132	ASN-131	ASN-154	ASN-293	ASN-292	ASN-172	LEU-135
LEU-134	LEU-133	LEU-156	LEU-295	LEU-294	LEU-174	ILE-148
ASP-145	ASN-144	ASP167	ASP-325	ASP-307	ASP-186	ASP-149

© 2018 by the authors. Submitted for possible open access publication under the terms and conditions of the Creative Commons Attribution (CC BY) license (http://creativecommons.org/licenses/by/4.0/).
